# Developmental endothelial locus-1 protects from hypertension-induced cardiovascular remodeling via immunomodulation

**DOI:** 10.1172/JCI126155

**Published:** 2022-03-15

**Authors:** Theresa Failer, Michael Amponsah-Offeh, Aleš Neuwirth, Ioannis Kourtzelis, Pallavi Subramanian, Peter Mirtschink, Mirko Peitzsch, Klaus Matschke, Sems M. Tugtekin, Tetsuhiro Kajikawa, Xiaofei Li, Anne Steglich, Florian Gembardt, Annika C. Wegner, Christian Hugo, George Hajishengallis, Triantafyllos Chavakis, Andreas Deussen, Vladimir Todorov, Irakli Kopaliani

**Affiliations:** 1 Department of Physiology and; 2 Institute for Clinical Chemistry and Laboratory Medicine, Medical Faculty Carl Gustav Carus, Technische Universität Dresden, Dresden, Germany.; 3 Hull York Medical School, York Biomedical Research Institute, University of York, York, United Kingdom.; 4 Department of Cardiac Surgery, Heart Center Dresden GmbH, Medical Faculty Carl Gustav Carus, Technische Universität Dresden, Dresden, Germany.; 5 University of Pennsylvania, Penn Dental Medicine, Department of Basic and Translational Sciences, Philadelphia, Pennsylvania, USA.; 6 Experimental Nephrology, Division of Nephrology, Department of Internal Medicine III, University Hospital Carl Gustav Carus, Technische Universität Dresden, Dresden, Germany.

**Keywords:** Cardiology, Inflammation, Cardiovascular disease, Hypertension

## Abstract

The causative role of inflammation in hypertension-related cardiovascular diseases is evident and calls for development of specific immunomodulatory therapies. We tested the therapeutic efficacy and mechanisms of action of developmental endothelial locus-1 (DEL-1), an endogenous antiinflammatory factor, in angiotensin II– (ANGII–) and deoxycorticosterone acetate–salt–induced (DOCA-salt–induced) cardiovascular organ damage and hypertension. By using mice with endothelial overexpression of DEL-1 (EC-Del1 mice) and performing preventive and interventional studies by injecting recombinant DEL-1 in mice, we showed that DEL-1 improved endothelial function and abrogated aortic adventitial fibrosis, medial thickening, and loss of elastin. DEL-1 also protected the mice from cardiac concentric hypertrophy and interstitial and perivascular coronary fibrosis and improved left ventricular function and myocardial coronary perfusion. DEL-1 prevented aortic stiffness and abolished the progression of hypertension. Mechanistically, DEL-1 acted by inhibiting **α**_v_**β**_3_ integrin–dependent activation of pro-MMP2 in mice and in human isolated aorta. Moreover, DEL-1 stabilized **α**_v_**β**_3_ integrin–dependent CD25^+^FoxP3^+^ Treg numbers and IL-10 levels, which were associated with decreased recruitment of inflammatory cells and reduced production of proinflammatory cytokines in cardiovascular organs. The demonstrated effects and immune-modulating mechanisms of DEL-1 in abrogation of cardiovascular remodeling and progression of hypertension identify DEL-1 as a potential therapeutic factor.

## Introduction

Hypertension is a major risk factor for development of cardiovascular diseases, which cause the highest number of deaths worldwide among noninfectious diseases (1). Currently, over one billion adults are affected by hypertension, and this number is projected to rise (1). Therefore, hypertension-caused cardiovascular diseases will remain a global health problem and a major socioeconomical health-care burden. During hypertension, the aorta undergoes fibrosis and hypertrophy along with degradation of elastic fibers and dilatation that lead to its stiffening (2). Aortic stiffening considerably increases afterload and leads to a progressive increase in systolic blood pressure (SBP), adversely affecting circulation in brain, kidney, and heart and by that increasing the incidence of stroke, renal failure, and myocardial infarction (3). Hypertension and aortic stiffness also contribute to the development of cardiac remodeling, resulting in left ventricular (LV) hypertrophy and fibrosis, which in turn adversely affect cardiac relaxation and coronary perfusion and also result in forced reduction of oxidative metabolism and increased reliance on glucose. Together, these pathological changes in the heart may result in cardiac failure (3, 4).

Although pathophysiological mechanisms of hypertension and associated cardiovascular diseases are not fully understood, excessive activation of the renin-angiotensin-aldosterone system and production of angiotensin II (ANGII) are critically involved (5). ANGII increases arterial pressure by inducing strong vasoconstriction and promotion of sodium and water reuptake (via aldosterone) from the renal tubules. While increased arterial pressure may contribute to cardiovascular organ damage, solid evidence indicates a critical direct role of inflammation in the development of hypertension and cardiovascular organ damage. Initial leukocyte recruitment of the innate immune system followed by T cell–driven inflammation and excessive IL-17 production mediate ANGII-induced cardiovascular remodeling and development of hypertension (6–12). In the deoxycorticosterone acetate–salt (DOCA-salt) model (hypervolemic hypertension induced by excessive renal salt retention), activated T cells and proinflammatory cytokines (e.g., IFN-γ, IL-17, and IL-18) are also involved in the pathogenesis of the hypertensive disorder and organ damage (13). Increase in arterial pressure and direct ANGII stimulation both promote proinflammatory CD4^+^ and CD8^+^ T cell immune response in cardiovascular tissues. A subset of proinflammatory CD4^+^ T cells, e.g., Th17 cells, produce IL-17, which in turn mediates cardiovascular remodeling. Gene knockout of IL-17 or neutralization with antibodies limits hypertension-induced inflammatory target organ damage and progression of hypertension (10–12, 14). The proinflammatory profile during ANGII-induced hypertension is accompanied by a destabilized and reduced antiinflammatory CD4^+^FoxP3^+^ Treg response along with a decrease in IL-10 production (14–18). This aggravates hypertension-induced inflammatory cardiovascular organ damage. Restoration of Treg response or supplementation of IL-10 protects from this damage both in vessels and heart (15–17, 19–21). Along with inflammation, cardiovascular remodeling during hypertension is accompanied by increased MMP2 activity (22–24). In aorta, MMP2 is released as a latent 72 kDa pro-MMP2 from smooth muscle cells (SMCs) and activated in an α_v_β_3_ integrin–dependent process by catalytic cleavage of the prodomain and activation to 62 kDa MMP2 (25). While α_v_β_3_ integrin engages pro-MMP2 on the cell membrane, furin activated membrane type 1 MMP (MT1-MMP) catalytically cleaves it (25). We previously demonstrated that activation of pro-MMP2 in aorta is endothelium/endothelin-1 (ET1) dependent. Endothelium-released ET1 regulates furin activity in SMCs, which in turn control MT1-MMP activity and ultimately activation of latent pro-MMP2 (26, 27). In heart, ANGII also stimulates release of pro-MMP2 from cardiac fibroblasts and cardiomyocytes, followed by catalytic activation by MT1-MMP (25). MMP2 is capable of degrading elastic fibers in aorta, and its activity is critically associated with aortic remodeling and an increase in aortic stiffness (22). MMP2 activities also contribute to cardiac hypertrophy and fibrosis (22).

Current treatment strategies to limit cardiovascular damage during hypertension are aimed at lowering arterial pressure, especially by inhibition of the renin-angiotensin system by angiotensin-converting enzyme inhibitors or angiotensin type 1 antagonists (1). However, strong evidential implications of inflammation in hypertension-induced cardiovascular diseases call for testing novel antiinflammatory strategies as a new approach for better management of hypertension-induced cardiovascular or other organ damage along with lowering arterial pressure. In this regard, inhibition of leukocyte recruitment and stabilizing/increasing antiinflammatory Treg numbers are considered as potential antiinflammatory therapeutic approaches to limiting target organ damage and progression of hypertension.

Developmental endothelial locus-1 (DEL-1) is an endogenous antiinflammatory glycoprotein expressed and secreted by many cell types, including endothelial cells (28–30). DEL-1 is highly expressed in immune-privileged organs, with a relatively low expression in cardiovascular tissues (28, 31, 32). Previously, we have shown that DEL-1 inhibits leukocyte recruitment and, most importantly, IL-17–dependent pathologies (28, 29, 33, 34). Its efficacy has been tested in IL-17–mediated inflammatory bone loss in mice and primates as well as in a mouse model of multiple sclerosis (28, 33, 34). Besides leukocyte recruitment, DEL-1 exerts its immunomodulatory actions via resolution of inflammation by efferocytosis and induction of antiinflammatory Treg response (35, 36). The antirecruitment action of DEL-1 is mediated via inhibition of β_2_ integrin/ICAM interaction, whereas efferocytosis and induction of Treg response are mediated via interaction of DEL-1 and α_v_β_3_ integrin by its RGD sequence in the second EGF repeat (29, 37). Thus, DEL-1 can resolve IL-17–mediated pathologies, such as inflammatory bone loss and multiple sclerosis, by suppressing inflammation with various antiinflammatory actions that depend on interaction of DEL-1 with integrins. However, whether the antiinflammatory properties of DEL-1 can prevent hypertension-related cardiovascular organ damage has not, to our knowledge, been hitherto addressed. Furthermore, it is unknown whether additional immunomodulatory actions of DEL-1 can be critical for prevention of organ damage during hypertension. In that regard, because DEL-1 interacts with α_v_β_3_ integrin, it is of particular interest to investigate whether DEL-1 interferes with the activation of pro-MMP2. Therefore, we investigated the efficacy and mechanisms of action of DEL-1 in ANGII- and DOCA-salt–induced inflammatory cardiovascular remodeling and progression of hypertension. For this, we performed experiments in a mouse model overexpressing endothelium-specific DEL-1 and, to obtain interventional proof, performed 3 different therapeutic studies by application of recombinant soluble DEL-1 in WT mice complemented with multiple human and mouse cell culture and isolated tissue experiments.

## Results

### DEL-1 overexpression protects from ANGII-induced aortic and cardiac structural and metabolic remodeling.

**Figure 1:**
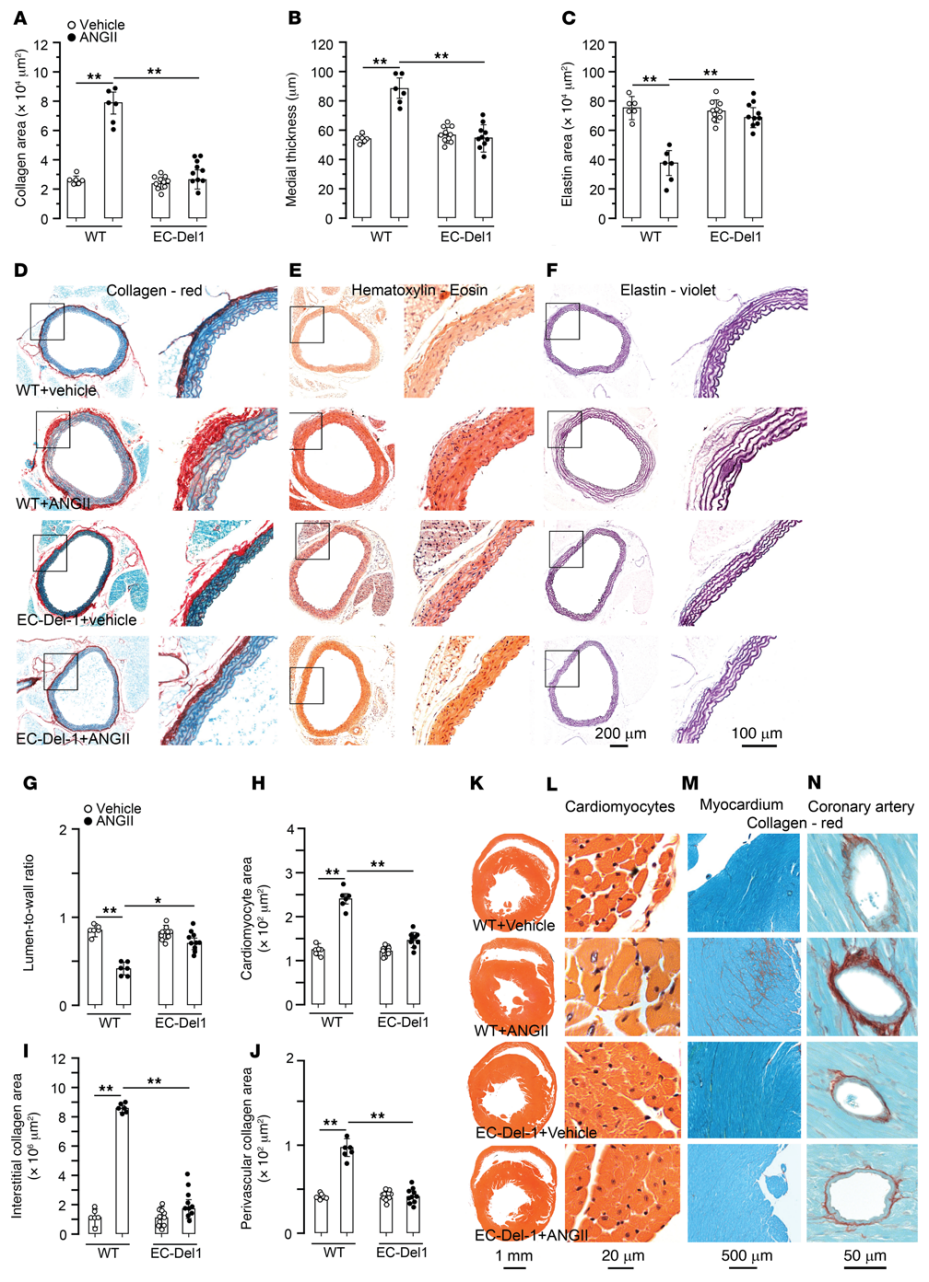
EC-Del1 mice are protected from ANGII-induced cardiovascular remodeling. 1.5 mg/kg/d ANGII or vehicle was infused in EC-Del1 and WT littermates for 4 weeks. Histological stainings and analysis of aortic adventitial collagen (**A** and **D**), medial wall thickness (**B** and **E**), and medial elastin (**C** and **F**) on the 28th day of ANGII or vehicle infusion. Histological stainings and analysis of cardiac lumen-to-wall ratio (**G** and **K**), cardiomyocyte cross-sectional area (**H** and **L**), interstitial (**I** and **M**) and perivascular coronary (**J** and **N**) collagen (*n* = 6–10 mice per group). Data are represented as mean ± SEM. *P < 0.05; **P < 0.01, 2-way ANOVA with Bonferroni’s post hoc test to adjust for multiple comparisons.

Major parameters of aortic remodeling, such as adventitial collagen, medial thickness, and elastin content, were assessed after 4 weeks of infusions of ANGII or vehicle. After ANGII infusion, WT mice had higher adventitial collagen (*P* < 0.01; Figure 1, A and D) and medial thickness (*P* < 0.01; Figure 1, B and E) along with lower elastin area (*P* < 0.01; Figure 1, C and F) compared with vehicle-infused WT mice. Compared with ANGII-infused WT mice, mice with endothelial overexpression of DEL-1 (EC-Del1 mice) had less adventitial collagen (*P* < 0.01; Figure 1, A and D) and lower medial thickness (*P* < 0.01; Figure 1, B and E) as well as more elastin (*P* < 0.01; Figure 1, C and F) after ANGII infusion. Cardiac remodeling was evaluated by assessment of LV lumen-to-wall ratio and cross-sectional area of cardiomyocytes as well as interstitial and perivascular collagen depositions. Compared with those receiving vehicle, ANGII-infused WT mice developed cardiac remodeling, resulting in decreased LV lumen-to-wall ratio (*P* < 0.01; Figure 1, G and K) and increased cardiomyocyte area (*P* < 0.01; Figure 1, H and L) along with profound deposition of interstitial (*P* < 0.01; Figure 1, I and M) and perivascular collagen (*P* < 0.01; Figure 1, J and N). Compared with ANGII-infused WT mice, EC-Del1 mice had higher LV lumen-to-wall ratio (*P* < 0.01; Figure 1, G and K) and lower cardiomyocyte area (*P* < 0.01; Figure 1, H and L) as well as less interstitial (*P* < 0.01;Figure 1, I and M) and perivascular collagen deposition (*P* < 0.01; Figure 1, J and N) after ANGII infusion.

**Figure 2:**
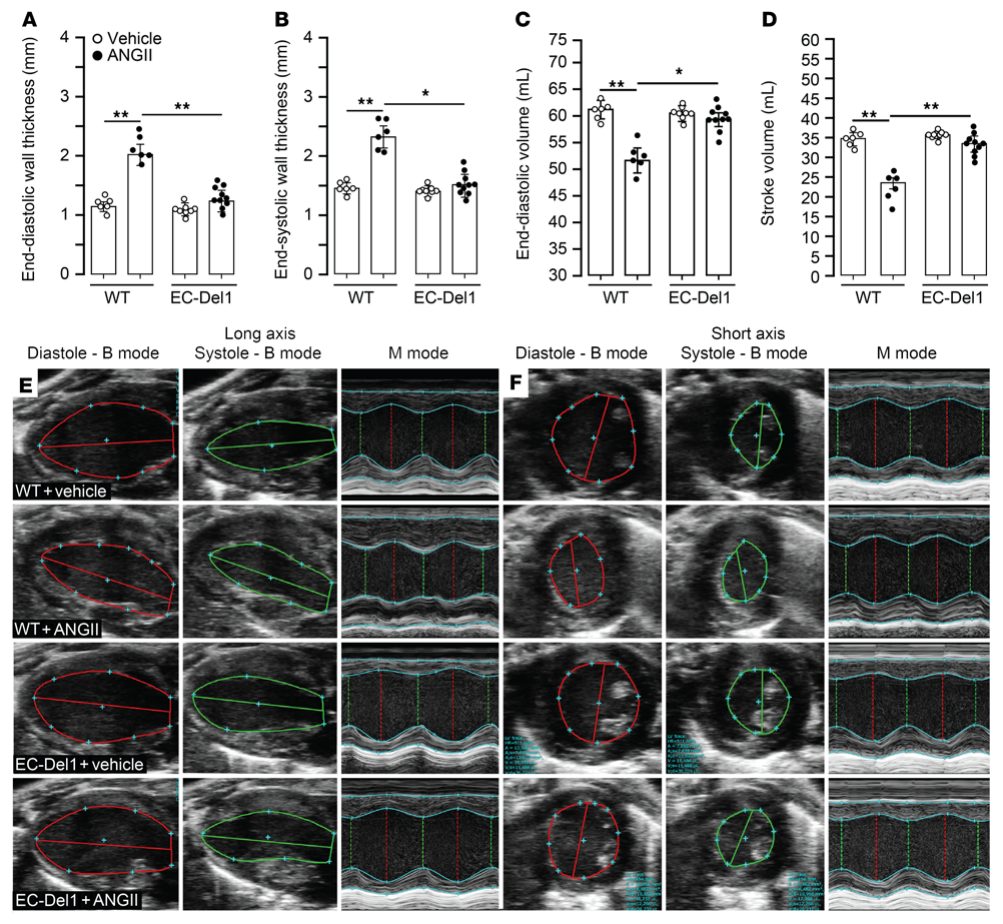
EC-Del1 mice are protected from ANGII-induced concentric cardiomyopathy. Echocardiographic evaluation and analysis of structural and hemodynamic parameters of concentric cardiomyopathy, cardiac end-diastolic (**A**) and end-systolic wall thickness (**B**), and end-diastolic (**C**) and stroke volumes (**D**). Representative echocardiographic images of B and M modes of long (**E**) and short axes (**F**). The parameters of concentric cardiomyopathy were analyzed in long axis (*n* = 6–10 mice per group). Data are represented as mean ± SEM. *P < 0.05; **P < 0.01, 2-way ANOVA with Bonferroni’s post hoc test to adjust for multiple comparisons.

**Figure 3:**
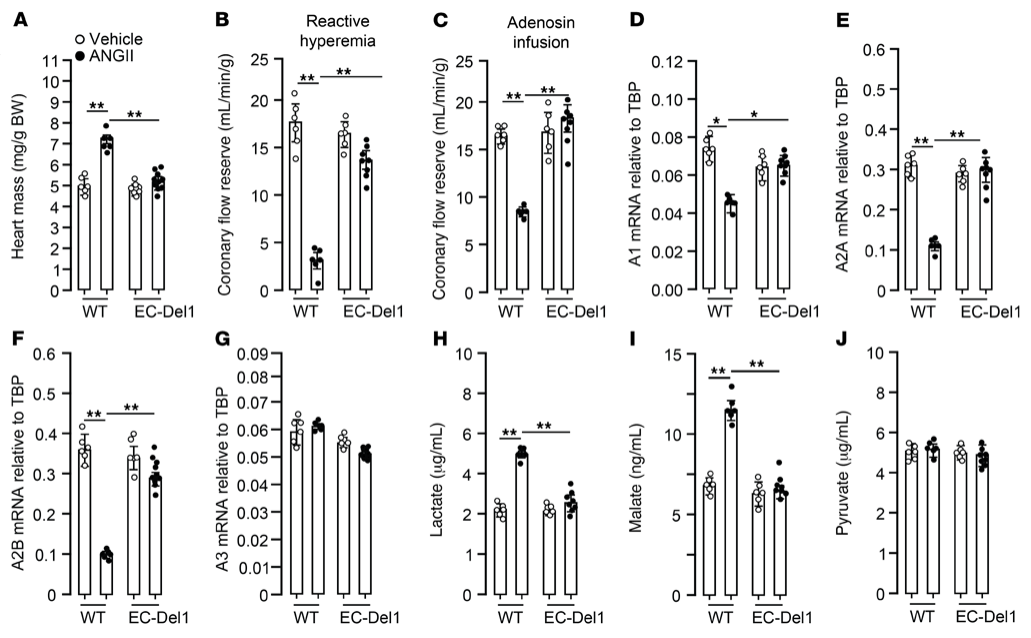
EC-Del1 mice are protected from ANGII-induced worsening of coronary flow and metabolic adaptation. Heart mass after 28 days of ANGII infusion (**A**) and coronary flow reserve assessed after flow stop (**B**) or adenosine infusion (**C**). mRNA levels of cardiac adenosine receptors A1, A2A, A2B, and A3 (**D**–**G**). Metabolites of glycolysis, lactate (**H**) and malate (**I**) along with pyruvate (**J**) (*n* = 6–10 mice per group). Data are represented as mean ± SEM. *P < 0.05; **P < 0.01, 2-way ANOVA with Bonferroni’s post hoc test to adjust for multiple comparisons.

Cardiac function was further evaluated and compared between groups with echocardiography and isolated perfused heart experiments. Compared with those receiving vehicle, ANGII-infused WT mice showed typical characteristics of pathological concentric hypertrophic cardiomyopathy expressed by increase in LV wall thickness during diastole (*P* < 0.01; Figure 2, A, E, and F) and systole (*P* < 0.01; Figure 2, B, E, and F) along with decreased end-diastolic (*P* < 0.01; Figure 2C) and stroke volumes (*P* < 0.01; Figure 2D). Compared with ANGII-infused WT mice, EC-Del1 mice showed lower LV wall thickness during diastole (*P* < 0.01; Figure 2, A, E, and F) and systole (*P* < 0.05; Figure 2, B, E, and F) along with higher end-diastolic (*P* < 0.05; Figure 2C) and stroke volumes (*P* < 0.01; Figure 2D). Compared with those receiving vehicle, ANGII-infused WT mice showed an increase in heart mass (*P* < 0.01; Figure 3A) and reduction in coronary flow reserve in response to a flow stop (*P* < 0.01; Figure 3B) and adenosine infusion (*P* < 0.01; Figure 3C). This was accompanied by a decrease in mRNA levels of adenosine type 1 (*P* < 0.01; Figure 3D), 2a (*P* < 0.01; Figure 3E), and 2b (*P* < 0.01; Figure 3F), but not type 3 (Figure 3G) receptors. We furthermore assessed cardiac metabolic adaptation after ANGII infusion by evaluation of glucose metabolism. For this, effluents from perfused hearts were collected for assessment of several metabolites. Compared with hearts from animals receiving vehicle, hearts of ANGII-infused WT mice showed higher lactate (*P* < 0.01; Figure 3H) and malate (*P* < 0.01; Figure 3I), whereas ANGII-infused EC-Del1 mice had lower lactate (*P* < 0.01; Figure 3H) and malate (*P* < 0.01; Figure 3I). Pyruvate did not differ between the groups (Figure 3J).

### DEL-1 overexpression protects from ANGII-induced progression of hypertension and endothelial dysfunction.

**Figure 4:**
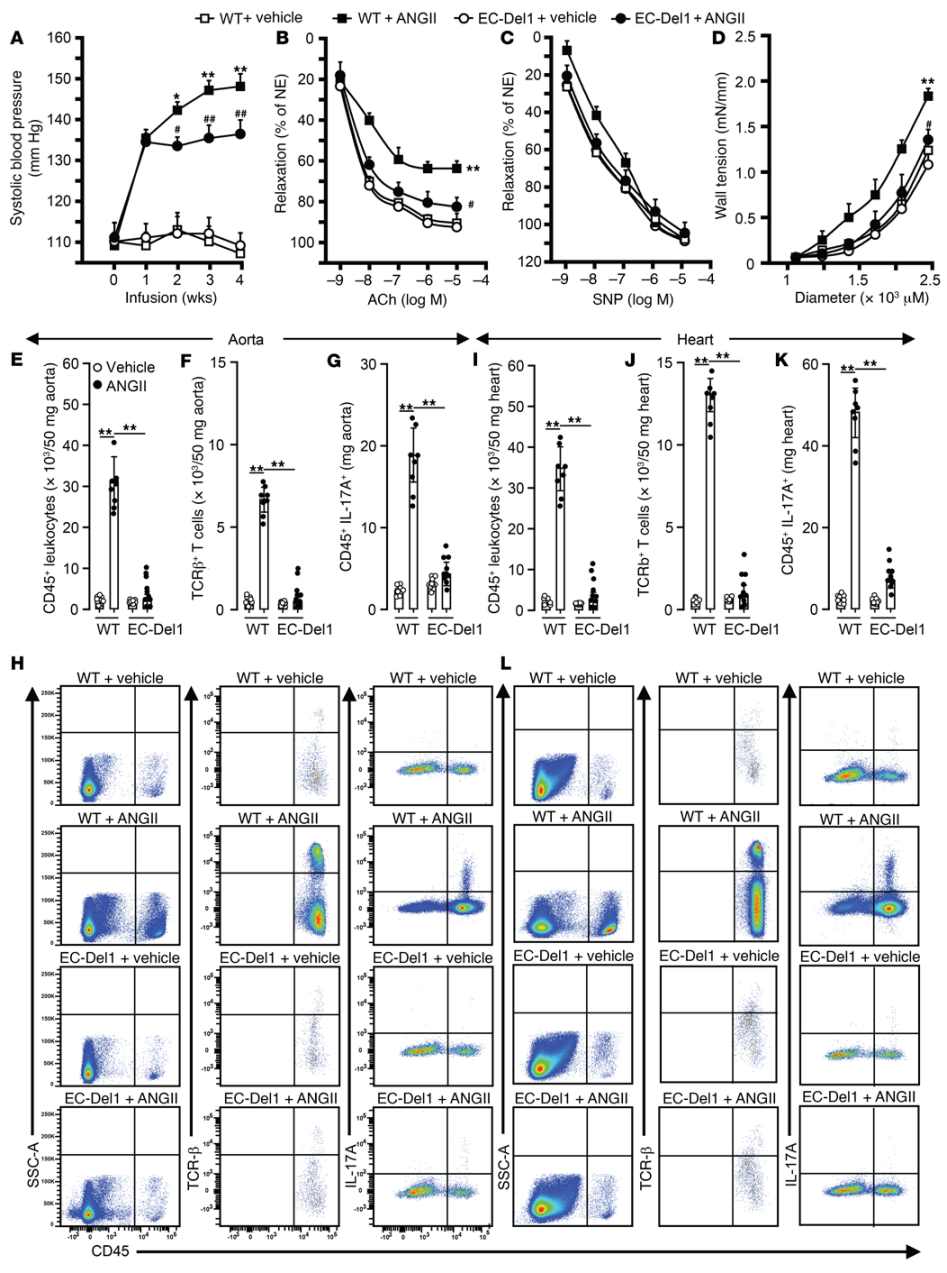
EC-Del1 mice are protected from ANGII-dependent progression of hypertension, arterial stiffness, and cardiovascular inflammation. SBP (**A**), ACh-mediated endothelium-dependent and -independent aortic relaxations (**B** and **C**), and aortic passive tension development (**D**) after 4 weeks of ANGII or vehicle infusion. FACS diagrams and plots of CD45^+^ leukocytes, TCR-β^+^ T cells, and CD45^+^IL-17A^+^ cells in aorta (**E**–**H**) and heart (**I**–**L**) (**A**, *n* = 12; **B**–**D**, *n* = 10; **E**–**L**, *n* = 8–10 mice per group). Data are represented as mean ± SEM. *P < 0.05 (vs. WT+vehicle in **A**); ^#^*P* < 0.05 (vs. WT+ANGII in **A**–**D**); **P < 0.01 (vs. WT+vehicle in **A**–**D**); ^##^*P* < 0.05 (vs. WT+ANGII in **A**–**D**), 2-way ANOVA (panels **A**–**D** for repeated measures) with Bonferroni’s post hoc test to adjust for multiple comparisons. NE, Norepinephrine.

We next assessed the effects of DEL-1 on development of SBP during 4 weeks of ANGII infusions. Before ANGII infusion, both EC-Del1 and WT littermates had similar SBP (Figure 4A). After 1 week of ANGII infusion, SBP was increased (*P* < 0.01) similarly in both WT and EC-Del1 mice compared with vehicle-infused mice (Figure 4A). In the following weeks, SBP increased gradually in WT, but not in EC-Del1, mice, resulting in profound differences in SBP among the groups at weeks 2 (*P* < 0.05), 3 (*P* < 0.01), and 4 (*P* < 0.01). Endothelial function was tested with assessment of acetylcholine-mediated (ACh-mediated) endothelium-dependent aortic relaxation. ACh-mediated maximal aortic relaxation after ANGII infusion was 62% in WT and 81% in EC-Del1 mice (*P* < 0.01; Figure 4B). Vehicle-infused EC-Del1 and WT mice showed similar, approximately 90%, ACh-mediated aortic relaxation. Maximum sodium nitroprusside–mediated (SNP-mediated) endothelium-independent relaxation was not different between groups (Figure 4C). We also assessed aortic stiffness by evaluating the diameter-tension relationship in isolated aorta in a passive state. With a gradual increase in diameter, wall tension increased in all groups (Figure 4D). However, the wall tension was significantly lower in ANGII-infused EC-Del1 mice compared with WT controls (*P* < 0.05, Figure 4D).

### DEL-1 overexpression suppresses inflammation in aorta and heart.

Studies have shown that ANGII infusion promotes T cell inflammation and IL-17–mediated aortic and cardiac remodeling along with progression of hypertension (6–12). We therefore investigated to determine whether the protective effects of DEL-1 on ANGII-induced cardiovascular remodeling and development of hypertension are associated with DEL-1–mediated inhibition of IL-17–dependent inflammation. To this end, we assessed the numbers of CD45^+^ leukocytes, TCR-β^+^ T cells, and CD45^+^IL-17^+^ leukocytes in aorta and heart. Compared with what occurred with vehicle, ANGII infusion profoundly increased CD45^+^ leukocytes, TCR-β^+^ T cells, and CD45^+^IL-17^+^ leukocytes in aortas and hearts of WT mice (*P* < 0.01, Figure 4, E–L). Aortas and hearts of EC-Del1 mice had significantly fewer CD45^+^ leukocytes, TCR-β^+^ T cells, and CD45^+^IL-17^+^ leukocytes after ANGII infusion compared with WT controls (*P* < 0.01, Figure 4, E–L).

### DEL-1 overexpression inhibits activation of MMP2 in aorta and heart.

**Figure 5:**
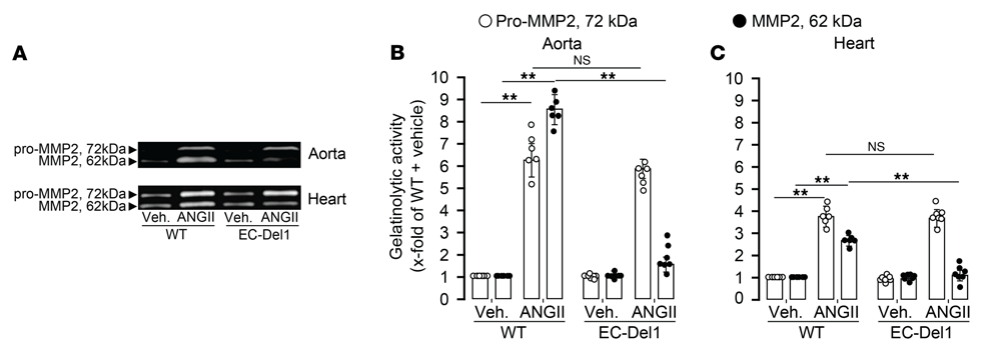
EC-Del1 mice have low MMP2 activity in cardiovascular tissues. Levels of latent pro-MMP2 and active MMP2 in aorta (**A** and **B**) and heart (**A** and **C**) after 4 weeks of ANGII or vehicle (Veh.) infusion (*n* = 6–7 mice per group). Data are represented as mean ± SEM. **P < 0.01, 2-way ANOVA with Bonferroni’s post hoc test to adjust for multiple comparisons.

Besides inflammation, increased levels of MMP2 critically contribute to aortic and cardiac remodeling in hypertension (23, 24). Based on this, we tested MMP2 activity in aorta and heart. ANGII-infused WT mice showed an approximately 6-fold increase in latent pro-MMP2 and an approximately 8-fold increase in active MMP2 in aorta compared with vehicle-infused WT mice (*P* < 0.01; Figure 5, A and B). ANGII-infused EC-Del1 mice showed a similar increase in latent pro-MMP2, but did not show the increase in active MMP2 compared with WT mice (Figure 5, A and B). In hearts, ANGII-infused WT mice demonstrated an approximately 4-fold increase in latent pro-MMP2 and an approximately 3-fold increase in active MMP2 (*P* < 0.01), whereas EC-Del1 mice showed a similar increase in latent pro-MMP2, but no increase in active MMP2 (Figure 5, A and C).

### DEL-1 overexpression protects from ANGII-induced increase in profibrotic and proinflammatory profiles in aorta and heart.

In addition to assessment of fibrosis and inflammation in aorta and heart using histology, we quantified mRNA levels for multiple profibrotic markers in these organs. These markers are presented in Supplemental Figure 1, A–O (aorta), and Supplemental Figure 2, A–O (heart) (supplemental material available online with this article; https://doi.org/10.1172/JCI126155DS1). Profibrotic markers in aorta, such as collagen 1 and 3 and fibronectin, were increased in ANGII-infused WT mice compared with those receiving vehicle (*P* < 0.01; Supplemental Figure 1, A–C). In heart, both the profibrotic markers and markers of hypertrophy, such as Myh-7, mBNP, and actin, were increased and serca decreased (*P* < 0.01; Supplemental Figure 2, A–F). ANGII-infused EC-Del1 mice showed lower mRNA levels of aortic collagen 1 and 3 and fibronectin compared with WT controls (*P* < 0.01; Supplemental Figure 1, A–C). In heart, these mice also had lower mRNA levels of Myh-7, mBNP, and actin along with more serca (*P* < 0.01; Supplemental Figure 2, A–F). Furthermore, mRNA and/or protein levels of proinflammatory factors (IL-17, IL-6, ICAM1, VCAM1, TGF-β1, INF-γ, TNF-α) were assessed and found to be increased in ANGII-infused WT mice compared with those receiving vehicle in both aorta (*P* < 0.01; Supplemental Figure 1, E–N) and heart (*P* < 0.01, Supplemental Figure 2, G–M). ANGII-infused EC-Del1 mice, however, showed lower mRNA and protein levels of these proinflammatory factors both in aorta (*P* < 0.01; Supplemental Figure 1, E–N) and heart (*P* < 0.01, Supplemental Figure 2, G–M). Assessment of antiinflammatory IL-10 revealed that ANGII-infused EC-Del1 mice had higher levels of IL-10 than WT controls in both aorta (*P* < 0.05; Supplemental Figure 1N) and heart (*P* < 0.01; Supplemental Figure 2N). We also quantified mRNA levels of DEL-1, which was 3 to 4 times higher in aorta (*P* < 0.01; Supplemental Figure 1O) and heart (*P* < 0.01; Supplemental Figure 2O) of EC-Del1 mice, respectively. ANGII infusion did not change mRNA levels of DEL-1.

### Recombinant DEL-1 prevents cardiovascular organ damage when injected before established hypertension.

**Figure 6:**
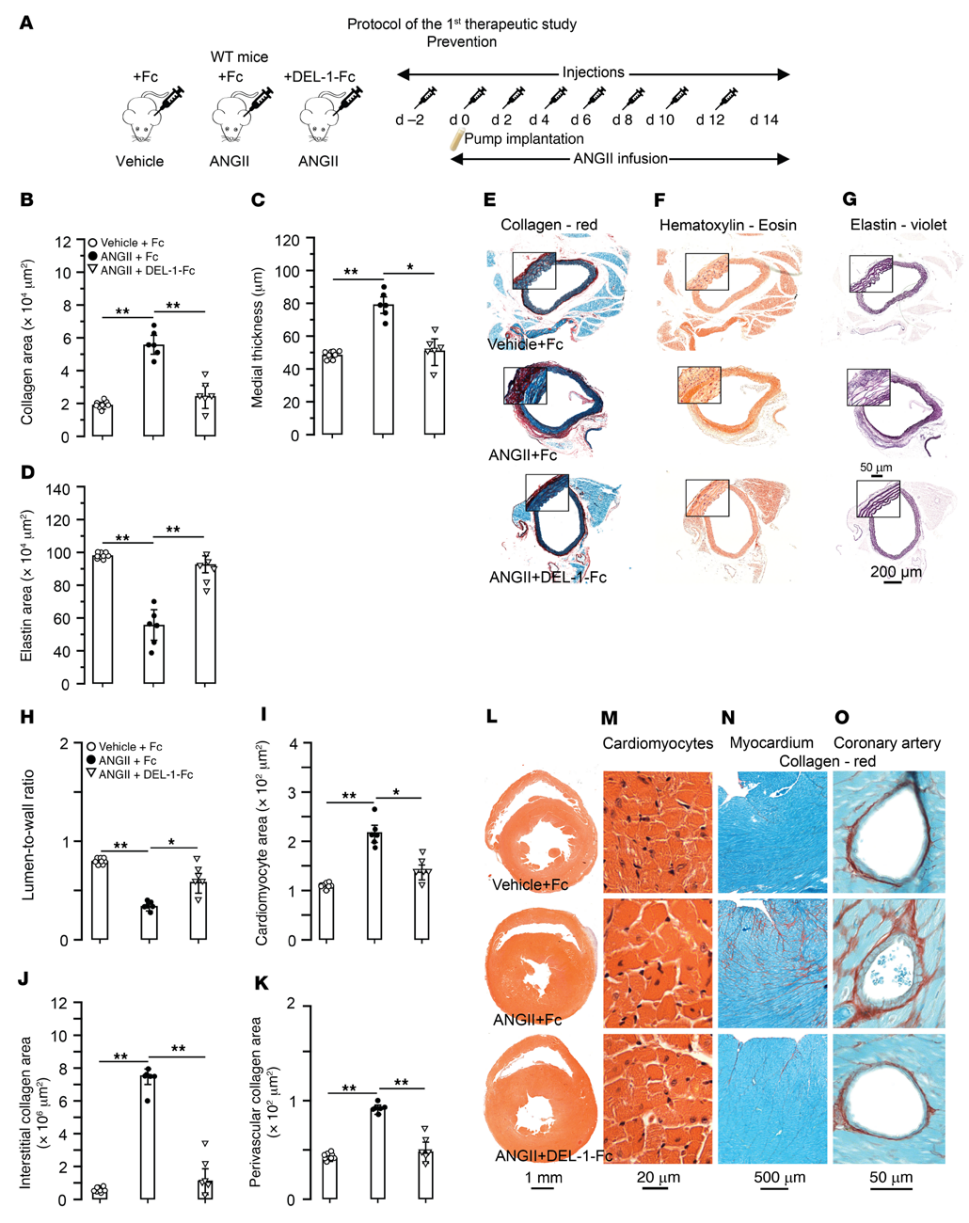
Treatment with DEL-1–Fc starting before established hypertension prevents cardiovascular remodeling. (**A**) Two days before starting the ANGII infusion, 30 μg of DEL-1–Fc or Fc alone was injected into WT mice. Additional injections were performed at the day of ANGII pump implantations and every second day up to the 12th day of infusion. Histological staining and analysis of aortic adventitial collagen (**B** and **E**), medial wall thickness (**C** and **F**), and medial elastin (**D** and **G**). Histological staining and analysis of LV lumen-to-wall ratio (**H** and **L**), cardiomyocyte cross-sectional area (**I** and **M**), and interstitial (**J** and **N**) and perivascular coronary (**K** and **O**) collagen (*n* = 6–8 mice per group). Data are represented as mean ± SEM. *P < 0.05; **P < 0.01, 1-way ANOVA with Bonferroni’s post hoc test to adjust for multiple comparisons.

In the prevention study, we tested efficacy of DEL-1 in protection from ANGII-induced cardiovascular damage. For this, recombinant DEL-1–Fc or Fc protein as a control was first injected in WT mice 2 days before ANGII infusion and then every other day over 2 weeks of infusion with ANGII or vehicle (Figure 6A). Fc-treated mice infused with ANGII demonstrated aortic remodeling with an increase in adventitial collagen (P < 0.01; Figure 6, B and E) and medial thickness (*P* < 0.01; Figure 6, C and F) and a decrease in elastin area (*P* < 0.01; Figure 6, D and G) compared with vehicle-infused mice. Compared with Fc-treated mice, mice treated with DEL-1–Fc were completely protected from the effects of ANGII, resulting in decreased adventitial collagen (*P* < 0.01; Figure 6, B and E) and medial thickness (*P* < 0.05; Figure 6, C and F) along with an increase in elastin area (*P* < 0.01; Figure 6, D and G). Fc-treated mice infused with ANGII also developed cardiac remodeling with decreased LV lumen-to-wall ratio (*P* < 0.01; Figure 6, H and L) and increased LV cardiomyocyte cross-sectional area (*P* < 0.01; Figure 6, I and M) and interstitial (*P* < 0.01; Figure 6, J and N) and perivascular coronary fibrosis (*P* < 0.01; Figure 6, K and O). Treatment of ANGII-infused mice with DEL-1–Fc, but not with Fc control, protected from cardiac remodeling, resulting in a higher LV lumen-to-wall ratio (*P* < 0.01; Figure 6, H and L) and a lower cardiomyocyte cross-sectional area (*P* < 0.05; Figure 6, I and M) as well as less interstitial (*P* < 0.01; Figure 6, J and N) and perivascular collagen (*P* < 0.01; Figure 6, K and O).

**Figure 7:**
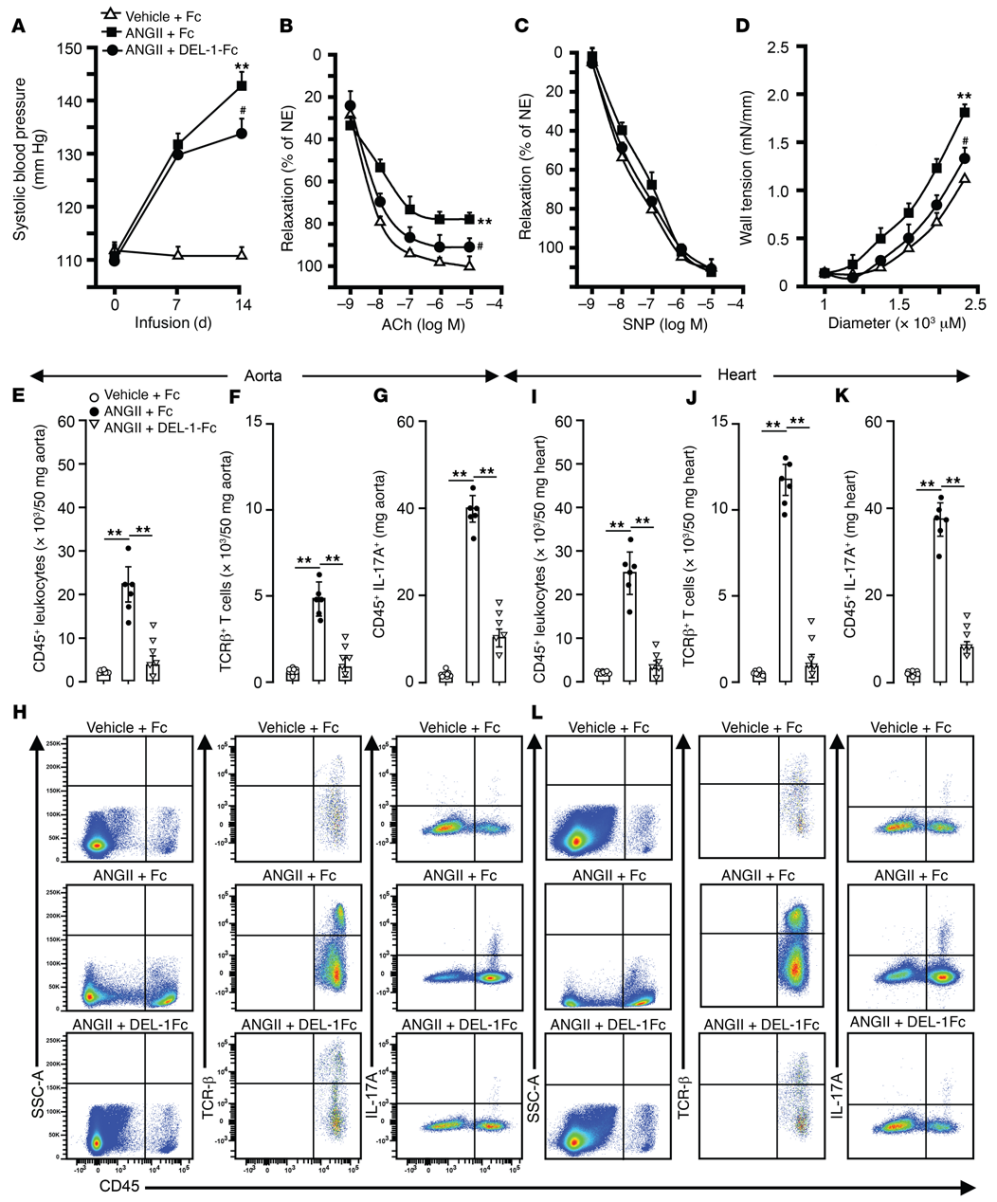
Treatment with DEL-1–Fc starting before established hypertension prevents progression of SBP, arterial stiffness, and cardiovascular inflammation. SBP (**A**), ACh-mediated endothelium-dependent and -independent aortic relaxations (**B** and **C**), and aortic passive tension development (**D**) after 2 weeks of ANGII or vehicle infusion with DEL-1–Fc or Fc treatment. FACS diagrams and plots of CD45^+^ leukocytes, TCR-β^+^ T cells, and CD45^+^IL-17A^+^ cells in aorta (**E**–**H**) and heart (**I**–**L**) (*n* = 6–8 mice per group). Data are represented as mean ± SEM. ^#^*P* < 0.05 (vs. WT+ANGII in **A**–**D**); **P < 0.01 (vs. WT+vehicle in **A**–**D**), 1-way ANOVA (panels **A**–**D** for repeated measures) with Bonferroni’s post hoc test to adjust for multiple comparisons.

After 2 weeks of ANGII infusion, DEL-1–Fc–treated mice had a lower (*P* < 0.05) SBP compared with mice treated with Fc (*P* < 0.05; Figure 7A). However, SBP did not differ in mice before or at the first week of ANGII infusion. ACh-mediated endothelium-dependent maximal aortic relaxation was approximately 70% in ANGII-infused Fc-treated mice and approximately 85% in mice treated with DEL-1–Fc (*P* < 0.05; Figure 7B). There was no difference in maximum SNP-mediated endothelium-independent relaxation (Figure 7C). Assessment of the diameter-tension relationship in isolated aorta in a passive state showed that wall tension increased in all groups with a gradual increase in diameter (Figure 7D). However, the wall tension was lower in ANGII-infused DEL-1–Fc–treated mice compared with that in mice treated with Fc alone (*P* < 0.05; Figure 7D).

Fc-treated mice showed profound increases in CD45^+^ leukocytes, TCR-β^+^ T cells, and CD45^+^IL-17^+^ leukocytes in aorta and heart after ANGII infusion compared with vehicle-infused Fc-treated control mice (*P* < 0.01; Figure 7, E–L). DEL-1–Fc–treated mice showed significantly lower counts of CD45^+^ leukocytes, TCR-β^+^ T cells, and CD45^+^IL-17^+^ leukocytes (*P* < 0.05; Figure 7, E–L) in aorta and heart after ANGII infusion compared with Fc-treated control mice.

**Figure 8:**
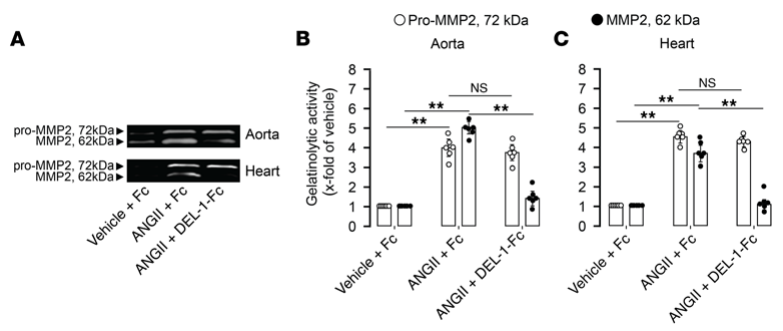
DEL-1–Fc–treated mice have low MMP2 activity in aorta and heart. Levels of latent pro-MMP2 and active MMP2 in aorta (**A** and **C**) and heart (**B** and **C**) (*n* = 6 mice per group). Data are represented as mean ± SEM. *P < 0.05; **P < 0.01, 1-way ANOVA with Bonferroni’s post hoc test to adjust for multiple comparisons.

Assessment of latent and active MMP2 showed that Fc-treated mice had increased pro- and active MMP2 levels in aorta (P < 0.01; Figure 8, A and B) and heart (*P* < 0.01; Figure 8, A and C) after ANGII infusion. Mice treated with DEL-1–Fc showed similar increases in latent pro-MMP2, but less active MMP2 in aorta (*P* < 0.01; Figure 8, A and B) and heart (*P* < 0.01; Figure 8, A and C). ANGII infusion increased mRNA levels of aortic profibrotic markers, such as collagen 1 and 3 and fibronectin, in Fc-treated mice (*P* < 0.01; Supplemental Figure 3, A–C), whereas these levels were lower in DEL-1–Fc–treated mice (*P* < 0.01; Supplemental Figure 3, A–C). In heart, mRNA levels of profibrotic and hypertrophy markers, such as collagen 1 and 3, Myh-7, mBNP, and actin, were increased and serca decreased in Fc-, but not in DEL-1–Fc–treated, mice (*P* < 0.01; Supplemental Figure 3, A–F). We furthermore assessed mRNA and protein levels of proinflammatory factors (IL-17, IL-6, ICAM1, VCAM1, TGF-β1, INF-γ, TNF-α) that were increased in Fc-treated mice after ANGII infusion compared with that in DEL-1–Fc–treated mice in both aorta (*P* < 0.01; Supplemental Figure 3, D–K) and heart (*P* < 0.01; Supplemental Figure 4, G–K). Levels of antiinflammatory IL-10 were higher in aorta (*P* < 0.05; Supplemental Figure 3L) and heart (*P* < 0.01; Supplemental Figure 4L) in ANGII-infused DEL-1–Fc–treated mice compared with those who received Fc treatment.

### Therapeutic intervention with recombinant DEL-1 after ANGII-induced established hypertension protects from cardiovascular organ damage via α_v_β_3_ integrin–dependent mechanisms.

**Figure 9:**
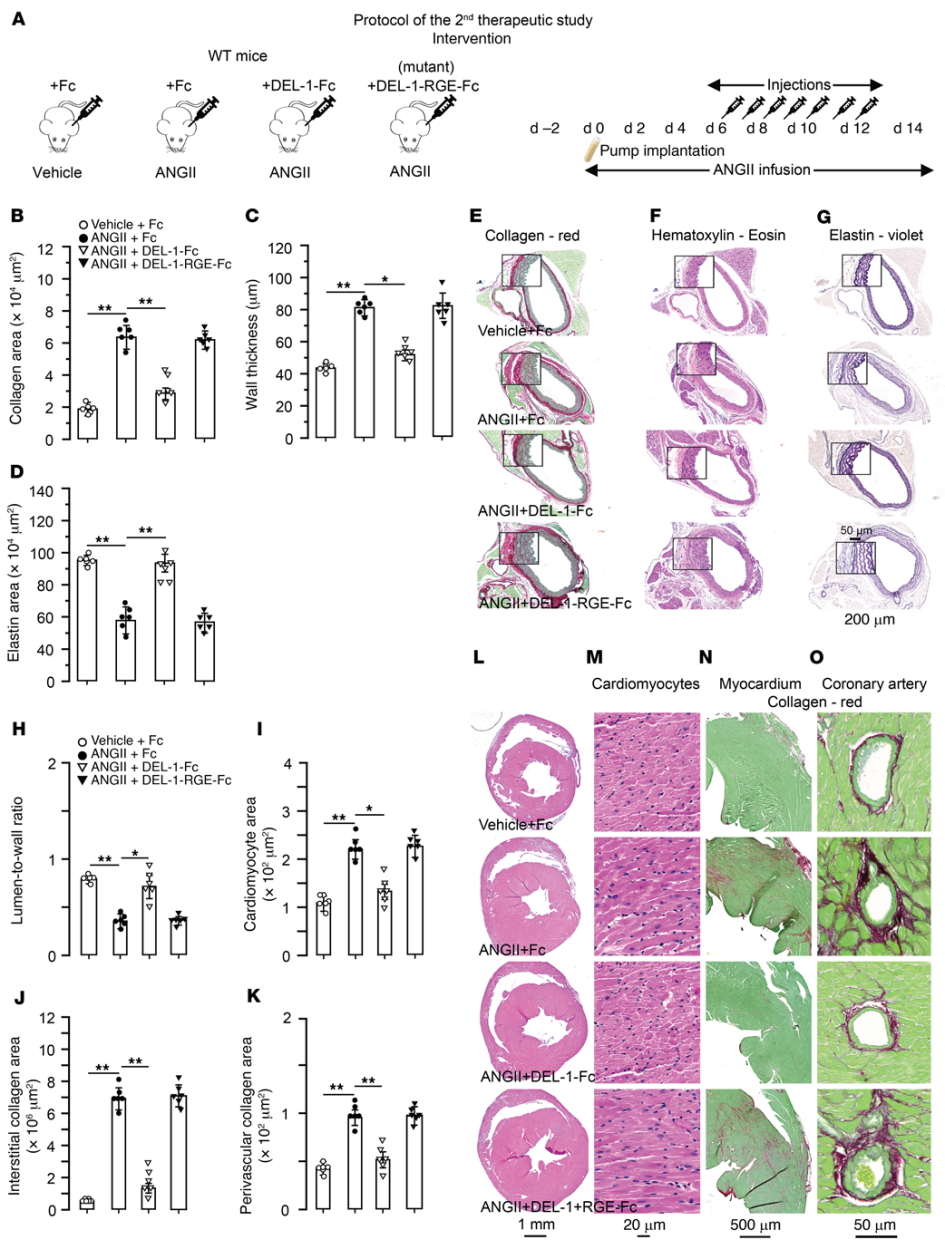
Treatment with DEL-1–Fc, but not DEL-1–RGE–Fc, starting after established hypertension abrogates cardiovascular remodeling in ANGII-induced hypertension. (**A**) On the sixth day after starting ANGII infusion, 20 μg of DEL-1–Fc, Del-RGE-Fc, or Fc alone was injected. Additional injections were performed every day up to the 12th day of ANGII infusion. Histological stainings and analysis of aortic adventitial collagen (**B** and **E**), medial wall thickness (**C** and **F**), and medial elastin (**D** and **G**). Histological stainings and analysis of cardiac lumen-to-wall ratio (**H** and **L**), cardiomyocyte cross-sectional area (**I** and **M**), and interstitial (**J** and **N**) and perivascular coronary (**K** and **O**) collagen (*n* = 6 mice per group). Data are represented as mean ± SEM. *P < 0.05; **P < 0.01, 1-way ANOVA with Bonferroni’s post hoc test to adjust for multiple comparisons.

In the second interventional study, we tested efficacy of DEL-1 in abrogation of ANGII-induced cardiovascular damage after established hypertension and investigated its mechanisms of action. For this, WT mice were infused with ANGII for 6 days before treatment with injections of either recombinant DEL-1–Fc, Fc protein as a control, or mutant DEL-1–RGE–Fc, which does not bind to α_v_β_3_ integrin, started. While ANGII infusion continued, treatments were undertaken every day up to day 12 of ANGII infusion (Figure 9A). We compared the effects on mice treated with Fc alone to those treated with DEL-1–Fc or DEL-1–RGE–Fc. Compared with those who received vehicle, Fc-treated mice infused with ANGII developed aortic remodeling with increased adventitial collagen (*P* < 0.01; Figure 9, B and E), increased medial thickness (*P* < 0.01; Figure 9, C and F), and decreased elastin (*P* < 0.01; Figure 9, D and G). Compared with Fc treatment, DEL-1–Fc treatment resulted in decreased adventitial collagen (*P* < 0.01; Figure 9, B and E) and medial thickness (*P* < 0.05; Figure 9, C and F) and increased elastin content (*P* < 0.01; Figure 9, D and G), whereas aortas of De-1–RGE–Fc–treated mice did not differ from those treated with Fc alone. Fc-treated mice infused with ANGII also developed cardiac remodeling with decreased LV lumen-to-wall ratio (*P* < 0.01; Figure 9, H and L) and increased LV cardiomyocyte cross-sectional area (*P* < 0.01; Figure 9, I and M) and interstitial (*P* < 0.01; Figure 9, J and N) and perivascular coronary fibrosis (*P* < 0.01; Figure 9, K and O). Treatment with DEL-1–Fc, but not with DEL-1–RGE–Fc, resulted in a higher LV lumen-to-wall ratio (*P* < 0.01; Figure 9, G and L) and a smaller cardiomyocyte cross-sectional area (*P* < 0.05; Figure 9, I and M) as well as less interstitial (*P* < 0.01; Figure 9, J and N) and perivascular collagen (*P* < 0.01; Figure 9, K and O).

**Figure 10:**
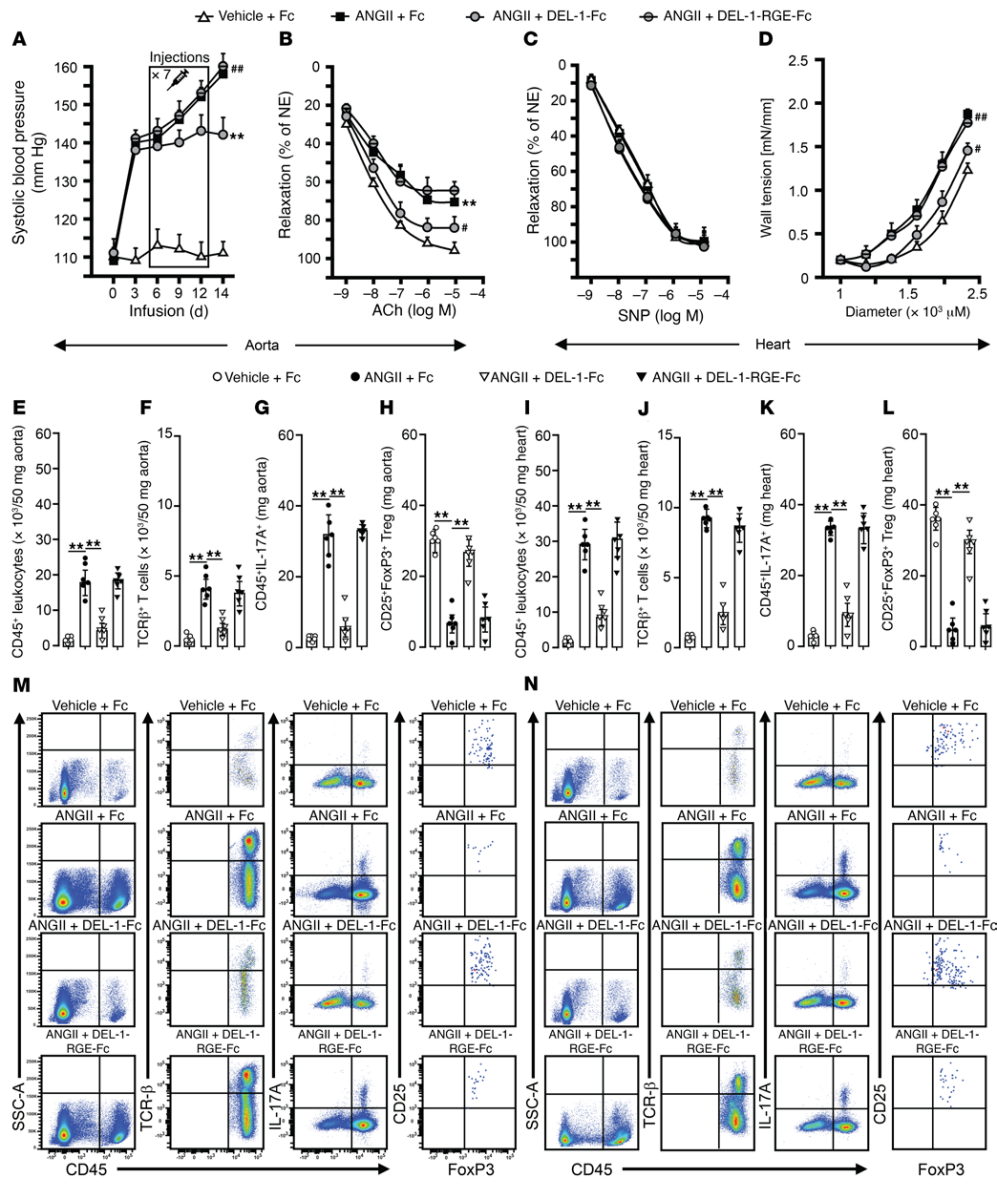
Treatment with DEL-1–Fc, but not DEL-1–RGE–Fc, starting after established hypertension abrogates progression of SBP, arterial stiffness, and cardiovascular inflammation and stabilizes antiinflammatory Treg numbers. SBP (**A**), endothelium-dependent (ACh) and -independent (SNP) aortic relaxations (**B** and **C**), and aortic passive tension development (**D**) after 2 weeks of ANGII or vehicle infusion with DEL-1–Fc, DEL-1–RGE–Fc, or Fc treatments. FACS diagrams and plots of CD45^+^ leukocytes, TCR-β^+^ T cells, and CD45^+^IL-17A^+^ and CD25^+^FoxP3^+^ Tregs in aorta (**E**–**H** and **M**) and heart (**I**–**L** and **N**) (*n* = 6 mice per group). Data are represented as mean ± SEM. ^#^*P* < 0.05 (vs. WT+ANGII in **B**–**D**); **P < 0.01 (vs. WT+vehicle in **A** and **B**); ^##^*P* < 0.05 (vs. WT+ANGII in **A** and **D**), 1-way ANOVA (panels **A**–**D** for repeated measures) with Bonferroni’s post hoc test to adjust for multiple comparisons.

Assessment of SBP showed that DEL-1–Fc–treated mice had a lower (*P* < 0.01) SBP compared with those treated with DEL-1–RGE–Fc or Fc (*P* < 0.05, Figure 10A) at the second week of ANGII infusion. However, SBP did not differ in mice before or at the sixth day of ANGII infusion, when the injections started. At the end of treatment periods, ACh-mediated endothelium-dependent maximal aortic relaxation was approximately 83% in DEL-1–Fc treated mice and approximately 72% and approximately 68% in DEL-1–RGE–Fc– or Fc-treated mice, respectively (*P* < 0.05; Figure 10B). There was no difference in maximum SNP-mediated endothelium-independent relaxation between groups (Figure 10C). Assessment of the diameter-tension relationship in isolated aorta showed that all groups responded with increases in wall tension that occurred by gradually increasing the diameter (Figure 10D). However, the wall tension was lower in ANGII-infused DEL-1–Fc–treated mice compared with DEL-1–RGE–Fc– or Fc-treated control mice (*P* < 0.05; Figure 10D).

**Figure 11:**
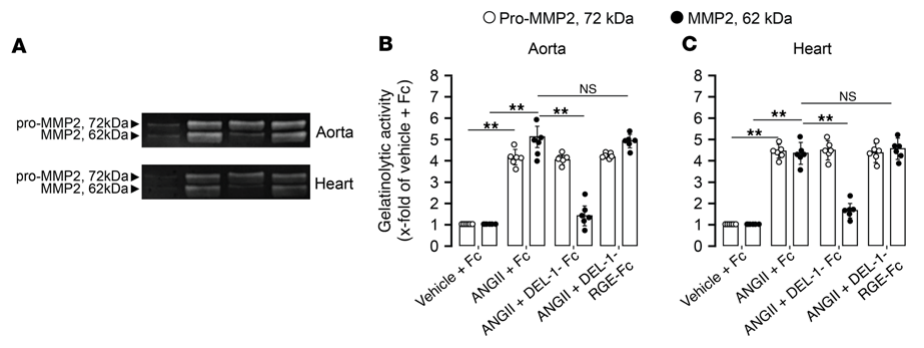
DEL-1–Fc–, but not DEL-1–RGE–Fc–treated, mice have low MMP2 activity in aorta and heart. Levels of latent pro-MMP2 and active MMP2 in aorta (**A** and **C**) and heart (**B** and **C**) (*n* = 6 mice per group). Data are represented as mean ± SEM. **P < 0.01, 1-way ANOVA with Bonferroni’s post hoc test to adjust for multiple comparisons.

ANGII-infused Fc-treated mice showed increases in CD45^+^ leukocytes, TCR-β^+^ T cells, and CD45^+^IL-17^+^ leukocytes along with decreases in CD25^+^FoxP3^+^ Tregs in aorta and heart compared with that in vehicle-infused Fc-treated mice (*P* < 0.01; Figure 10, E–N). DEL-1–Fc–treated mice infused with ANGII showed significantly lower counts of CD45^+^ leukocytes, TCR-β^+^ T cells, and CD45^+^IL-17^+^ leukocytes along with higher numbers of CD25^+^FoxP3^+^ Tregs in aorta and heart compared with DEL-1–RGE–Fc– or Fc-treated mice (*P* < 0.01; Figure 10, E–N). We further assessed the levels of latent and active MMP2 after ANGII infusion, which showed that Fc-treated mice had increased latent pro- and active MMP2 in aorta (*P* < 0.01; Figure 11, A and B) and heart (*P* < 0.01; Figure 11, A and C). Increases in latent pro-MMP2 were not different in mice treated with DEL-1–Fc, DEL-1–RGE–Fc, or Fc alone, whereas active MMP2 was lower in DEL-1–Fc than in DEL-1–RGE–Fc– or Fc–treated mice in both aorta (*P* < 0.01; Figure 11, A and B) and heart (*P* < 0.01; Figure 11, A and C). mRNA levels of aortic profibrotic markers, such as collagen 1 and 3 and fibronectin, were also less in DEL-1–Fc– than in DEL-1–RGE–Fc– or Fc-treated mice (*P* < 0.01; Supplemental Figure 5, A–C). Cardiac mRNA levels of profibrotic and hypertrophy markers, such as collagen 1 and 3, Myh-7, mBNP, and actin, were decreased and serca increased in DEL-1–Fc– compared with DEL-1–RGE–Fc– or Fc-treated mice (*P* < 0.01; Supplemental Figure 6, A–F). We further assessed mRNA and/or protein levels of proinflammatory factors (IL-17, IL-6, ICAM1, VCAM1, TGF-β1, INF-γ, TNF-α), and results showed that they were lower in DEL-1–Fc than DEL-1–RGE–Fc– or Fc-treated mice in both aorta (*P* < 0.01; Supplemental Figure 5, D–K) and heart (*P* < 0.01; Supplemental Figure 6, G–K). Levels of IL-10 were higher in ANGII-infused DEL-1–Fc–treated mice compared with those receiving DEL-1–RGE–Fc or Fc treatment in aorta (*P* < 0.05; Supplemental Figure 5L) and heart (*P* < 0.01; Figure 6L).

### Therapeutic intervention with recombinant DEL-1 after DOCA-salt–induced established hypertension protects from cardiovascular organ damage.

**Figure 12:**
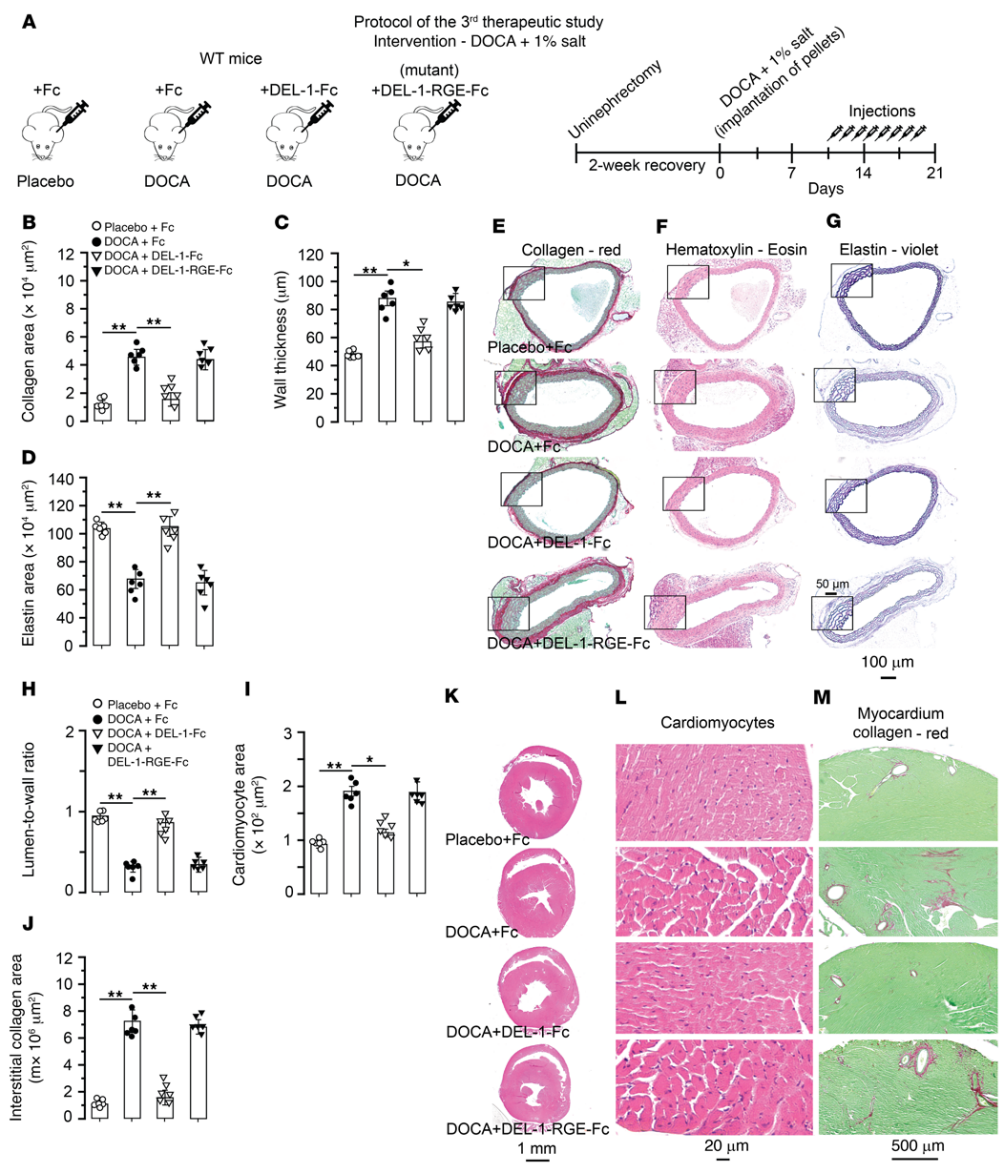
Treatment with DEL-1–Fc, but not DEL-1–RGE–Fc, starting after established hypertension abrogates cardiovascular remodeling in DOCA-salt hypertension. (**A**) On the tenth day after implantation of DOCA pellets, 20 μg of DEL-1–Fc, Del-RGE-Fc, or Fc alone was injected. A total of 8 injections up to the 18th day were performed. Histological stainings and quantification of aortic adventitial collagen (**B** and **E**), medial wall thickness (**C** and **F**), and medial elastin (**D** and **G**). Histological stainings and quantification of cardiac lumen-to-wall ratio (**H** and **K**), cardiomyocyte cross-sectional area (**I** and **L**), and interstitial (**J** and **M**) collagen (*n* = 6 mice per group). Data are represented as mean ± SEM. *P < 0.05; **P < 0.01, 1-way ANOVA with Bonferroni’s post hoc test to adjust for multiple comparisons.

**Figure 13:**
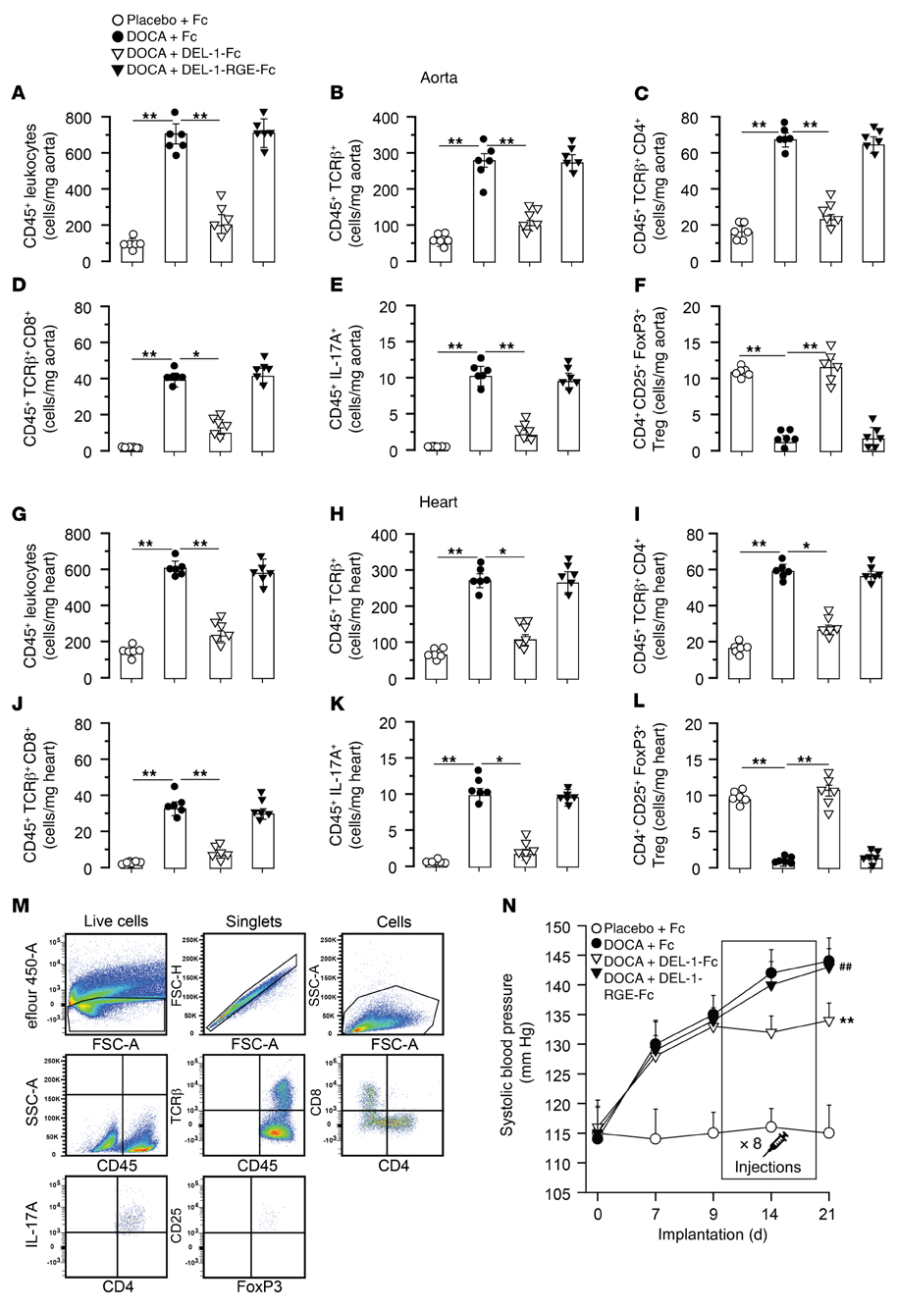
Treatment with DEL-1–Fc, but not DEL-1–RGE–Fc, starting after established DOCA-salt–induced hypertension abrogates progression of SPB and cardiovascular inflammation and stabilizes antiinflammatory Treg numbers. FACS diagrams of inflammatory cells in aorta (**A**–**F**) and heart (**G**–**L**). SBP development at up to 21 days of DOCA-salt hypertension (**N**). Representative gating strategy for CD45^+^, CD45^+^TCR-β^+^, CD45^+^TCR-β^+^CD4^+^, CD45^+^TCR-β^+^CD8^+^, CD4^+^IL-17A^+^, and CD4^+^CD25^+^FoxP3^+^ cells (**M**) (*n* = 6 mice per group). Data are represented as mean ± SEM. *P < 0.05; **P < 0.01, 1-way ANOVA (panel Z for repeated measures) with Bonferroni’s post hoc test to adjust for multiple comparisons.

Effects of DEL-1 were tested in another model of hypertension induced with DOCA-salt. In this third therapeutic study, injections started at the tenth day, with the last injection at the 18th of 21 days of DOCA-salt hypertension (Figure 12A). Histological assessments showed that DEL-1–Fc–treated mice had profoundly less aortic adventitial collagen (*P* < 0.01; Figure 12, B and E) and medial thickening (*P* < 0.05; Figure 12, C and F) with more elastin (*P* < 0.01; Figure 12, D and G) compared with Fc-treated mice. Assessment of cardiac parameters demonstrated that DEL-1–Fc–treated mice had higher LV lumen-to-wall ratio (*P* < 0.01; Figure 12, H and K) and less cardiomyocyte cross-sectional area (*P* < 0.05; Figure 12, I and L) and interstitial collagen (*P* < 0.01; Figure 12, J and M) compared with mice treated with Fc. Treatment with DEL-1–RGE–Fc did not affect aortic or cardiac remodeling and showed no difference from treatment with Fc (Figure 12, B–M). Numbers of CD45^+^, CD45^+^TCR-β^+^, CD45^+^TCR-β^+^CD4^+^, CD45^+^TCR-β^+^CD8^+^, and CD4^+^IL-17A^+^ cells in aorta (Figure 13, A–E) and heart (Figure 13, G–K) were lower in DEL-1–Fc–treated mice compared with mice treated with Fc alone. Treatment with DEL-1–Fc resulted in increased numbers of CD4^+^CD25^+^FoxP3^+^ Tregs compared with Fc treatment in both aorta (Figure 13F) and heart (Figure 13L). Injections of DEL-1–RGE–Fc did not affect numbers of inflammatory cells in aorta and heart and showed no difference compared with treatment with Fc (Figure 13, A–M). SBP did not differ in mice before injections on the seventh day of DOCA-salt hypertension (Figure 13N). After injection of DEL-1–Fc, SBP did not further increase in this group, whereas gradual increase in SBP was observed in DEL-1–RGE–Fc– and Fc-treated groups. Mice treated with DEL-1–Fc showed approximately 10 mm Hg lower SBP compared with mice treated with Fc or DEL-1–RGE–Fc on days 14 and 21. Mice implanted with a placebo pallet and treated with Fc remained normotensive.

### DEL-1 inhibits α_v_β_3_ integrin–dependent activation of latent pro-MMP2 in vascular tissue.

**Figure 14:**
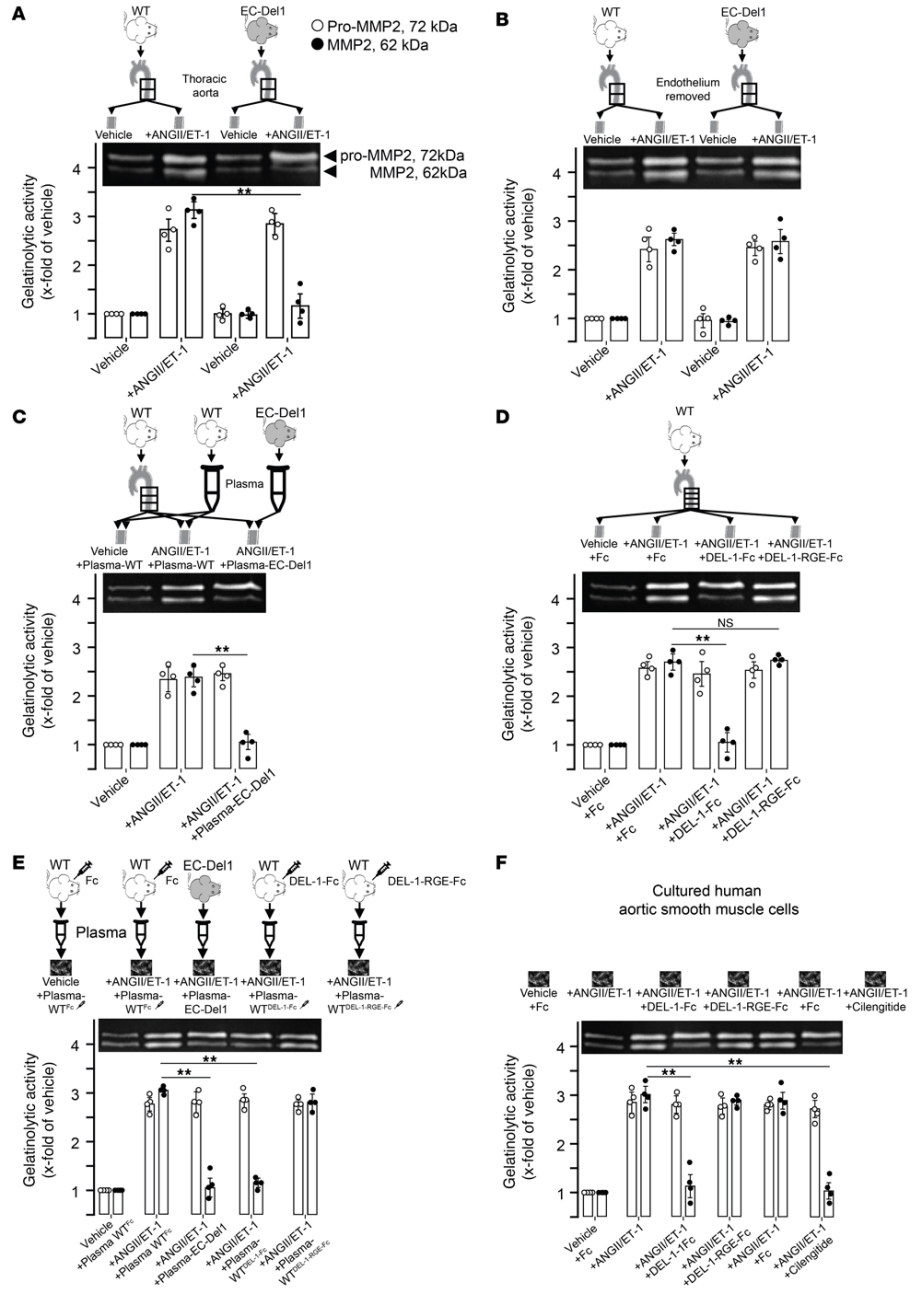
DEL-1 inhibits α_v_β_3_ integrin–dependent activation of latent pro-MMP2. Thoracic aorta was isolated from WT and EC-Del1 mice and stimulated with ANGII/ET1, followed by assessment of latent pro- and active MMP2 (**A**). Stimulation of isolated aorta from WT and EC-Del1 mice without endothelium (**B**). ANGII/ET1-stimulated aortas of WT mice pretreated with Fc, DEL-1–Fc, or DEL-1–RGE–Fc (**C**). ANGII/ET1–stimulated aortas of WT mice pretreated with plasma of EC-Del1 mice (**D**). Cultured human aortic SMCs pretreated with plasmas of WT mice injected with Fc, DEL-1–Fc, DEL-1–RGE–Fc, or plasma of EC-Del1 mice and then stimulated with ANGII/ET1 (**E**). Cultured human aortic SMCs pretreated with Fc, DEL-1–Fc, DEL-1–RGE–Fc, or pharmacological α_v_β_3_ integrin cilengitide and then stimulated with ANGII/ET1 (**F**) (**A**–**F** *n* = 4 independent experiments). Data are represented as mean ± SEM. **P < 0.01, 1-way ANOVA with Bonferroni’s post hoc test to adjust for multiple comparisons.

In several consecutive ex vivo experiments, we studied the role and mechanism of DEL-1 in inhibition of activation of latent pro-MMP2 in aorta and cultured human aortic SMCs (Figure 14). These cells were chosen because SMCs are the major source of MMP2 in aorta and α_v_β_3_ integrin on SMCs is critical in activation of latent pro-MMP2 (22, 25). In the first set of experiments, aortas were isolated from WT and EC-Del1 mice. followed by stimulation with ANGII/ET1 and assessment of latent pro- and active MMP2 (Figure 14A). Compared with use of vehicle, ANGII/ET1 stimulation increased latent pro-MMP2 in isolated aortas of both WT and EC-Del1 mice similarly (*P* < 0.01; Figure 14A), whereas active MMP2 was less in aortas of EC-Del1 mice (*P* < 0.01; Figure 14A). Because we employed endothelial-specific EC-Del1 mice, in the second set of experiments, we stimulated isolated aortas from WT and EC-Del1 mice after removal of endothelium (Figure 14B). Stimulation with ANGII/ET1 similarly increased both latent pro- and active MMP2 in isolated aortas of WT and EC-Del1 mice in the absence of endothelium (*P* < 0.01; Figure 14B), showing no difference in MMP2 in aortas of mice. In the third set of experiments, isolated aortas of WT mice were pretreated with plasmas of EC-Del1 or WT mice, followed by stimulation with ANGII/ET1. Isolated aortas pretreated with plasma from EC-Del1 mice showed less active MMP2 than aortas pretreated with plasma from WT mice (*P* < 0.01; Figure 14C). Treatment with plasma from EC-Del1 mice did not affect increases in latent pro-MMP2 (Figure 14C). In the fourth set of experiments, we studied the role of α_v_β_3_ integrin in DEL-1–mediated inhibition of activation of latent pro-MMP2 by pretreating isolated aortas of WT mice with Fc, DEL-1 Fc, or DEL-1–RGE–Fc and then stimulating with ANGII/ET1. Stimulation with ANGII/ET1 increased latent pro-MMP2 equally in isolated aortas treated with Fc, DEL-1 Fc, or DEL-1–RGE–Fc (*P* < 0.01; Figure 14D). However, active MMP2 was less in DEL-1–Fc–treated aorta, but not in aorta treated with DEL-1–RGE–Fc (*P* < 0.01; Figure 14D). In the fifth set of experiments, we studied the effects of treatment with plasmas from WT mice injected with Fc, DEL-1–Fc, or DEL-1–RGE–Fc on inhibition of activation of latent pro-MMP2 in cultured human aortic SMCs (Figure 14E). Treatment with plasma from Fc-injected mice did not inhibit ANGII/ET1-mediated increase in latent pro- and active MMP2. Treatment with plasmas from EC-Del1 mice did not affect latent pro-MMP2, but decreased active MMP2 (*P* < 0.01; Figure 14E). Plasmas from DEL-1–Fc– but not DEL-1–RGE–Fc–injected mice decreased active MMP2 without affecting latent pro-MMP2 (*P* < 0.01). In the final set of experiments, cultured human aortic SMCs were pretreated with recombinant DEL-1–Fc or DEL-1–RGE–Fc along with the pharmacological inhibitor of α_v_β_3_ integrin cilengitide, followed by stimulation with ANGII/ET1 (Figure 14F). Treatment with DEL-1–Fc or cilengitide decreased ANGII/ET1-induced active MMP2 without affecting latent pro-MMP2 (*P* < 0.01), whereas treatment with DEL-1–RGE–Fc or Fc did not affect the induction of active MMP2, thus conclusively implicating αvβ3 integrin as the target of DEL-1.

### DEL-1 inhibits α_v_β_3_ integrin–dependent activation of latent pro-MMP2 in human isolated aorta.

**Figure 15:**
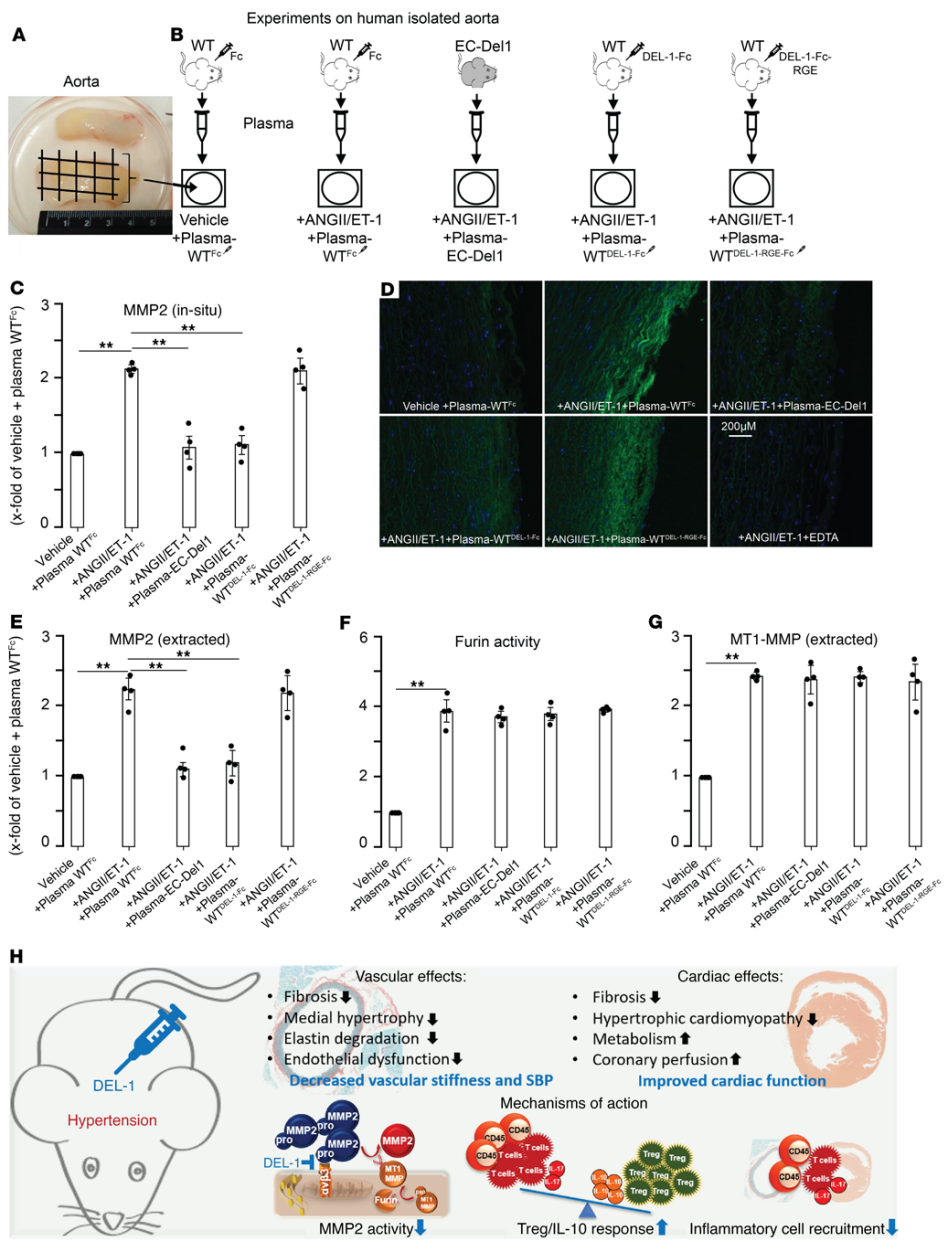
DEL-1 inhibits α_v_β_3_ integrin–dependent activation of latent pro-MMP2 in isolated human aorta. A piece of isolated human aorta, which was divided into smaller 5 × 5 mm^2^ pieces and incubated in tissue well plates, followed by pretreatment with plasmas of Fc-, DEL-1–Fc–, and DEL-1–RGE–Fc–injected mice or plasma of EC-Del1 mice and then stimulated with ANGII/ET1 (**A** and **B**). In situ gel zymography showing MMP2 activity in isolated human aorta (**C** and **D**). MMP2 (**E**), furin (**F**), and MT1-MMP (**G**) activity assays in extracts of isolated human aorta. Schematic summary of protective effects and antiinflammatory mechanisms of action of DEL-1 in ANGII-induced hypertension and cardiovascular remodeling (**H**) (**A**–**G**, *n* = 4 independent experiments). Data are represented as mean ± SEM. **P < 0.01, 1-way ANOVA with Bonferroni’s post hoc test to adjust for multiple comparisons.

Pieces of human isolated aorta were pretreated with plasma from Fc-, DEL-1–Fc–, and DEL-1–RGE–Fc–injected WT mice or with plasma from EC-Del1 mice (Figure 15, A and B). After pretreatment, aortas were stimulated with ANGII/ET1 or vehicle. In situ zymography on human isolated aorta showed that, compared with vehicle, ANGII/ET1 increased gelatinolytic activity of MMP2 in aortic sections pretreated with plasma from Fc-treated mice (Figure 15, C and D). Pretreatment with plasmas from EC-Del1 or DEL-1–Fc–treated mice resulted in inhibition of gelatinolytic activity, whereas pretreatment with plasma from DEL-1–RGE–Fc–treated mice did not inhibit gelatinolytic activity. Control pretreatment with EDTA resulted in complete inhibition of gelatinolytic activity of zinc-dependent MMP2.

Additionally, MMP2 was extracted from aortas and its in vitro activity was assessed. The results were similar to those seen with the in situ zymography. Compared with vehicle, MMP2 activity was higher in aortic sections pretreated with plasmas from Fc-injected mice and stimulated with ANGII/ET1 (Figure 15E). Pretreatment with plasmas from EC-Del1 or DEL-1–Fc–treated mice, but not from DEL-1–RGE–Fc–treated mice resulted in inhibition of MMP2 activity. We further assessed components of activation machinery of pro-MMP2, such as furin and MT1-MMP, which, in conjunction with α_v_β_3_ integrin, activate latent pro-MMP2 (25, 26). Treatment with various plasmas (from EC-Del1 mice or DEL-1–Fc–, DEL-1–RGE–Fc–, or Fc-treated mice) did not inhibit furin or MT1-MMP in human isolated aorta (Figure 15, F and G).

## Discussion

We demonstrate a protective function of DEL-1 in two models of hypertension-induced cardiovascular remodeling. Consistent findings from EC-Del1 mice with endothelial-specific DEL-1 overexpression and therapeutic intervention studies with injections of recombinant DEL-1 provide compelling evidence that DEL-1 protects from adverse remodeling both in aorta and heart and also hinders the progression of hypertension. We propose that the mechanisms of action of DEL-1 largely depend on α_v_β_3_ integrin–dependent effects. By interacting with the α_v_β_3_ integrin, DEL-1 inhibits activation of latent pro-MMP2 and stabilizes antiinflammatory Treg/IL-10 response, with consequent suppression of IL-17 production and proinflammatory cell recruitment in target organs.

Cardiovascular remodeling is a hallmark of hypertension. Vascular and cardiac remodeling interact in a complex manner so that they augment each other, leading to further augmentation of SBP and organ damage (2, 3). Therefore, control of arterial pressure and attenuation of cardiovascular remodeling by therapeutic intervention is of critical importance. We demonstrate that DEL-1 can prevent hypertension-induced aortic and cardiac remodeling as well as abrogate further progression of SBP. This is supported by results obtained from 4 different sets of animal experiments using genetic overexpression of endothelial DEL-1 and therapeutic injections of recombinant DEL-1–Fc before or after onset of hypertension. Findings from the EC-Del-1 model and what we believe to be the first therapeutic study demonstrate that DEL-1 abrogates progression of hypertension. Whereas SBP was similar in EC-Del1 or DEL-1–Fc–treated mice compared with their respective controls in the first week of hypertension induction, SBP did not further increase in EC-Del1 or DEL-1–Fc–treated mice at later times. Initial increase in SBP in the first week was merely a result of the vasoconstrictive effect in both models resulting in an increase in LV afterload (7, 10, 11). Further progression of SBP seen from second week of induction of hypertension can be attributed to aortic remodeling and stiffening and worsening of endothelial function, important parameters that augment systolic hypertension (5, 11). Stiffness of the aorta reduces its capacity to expand (Windkessel effect) during systole, decreases LV afterload, and dampens systolic pressure. Endothelial dysfunction causes a decrease in bioavailability of NO, which may increase peripheral vessel tone. Therefore, arterial stiffening and endothelial dysfunction result in worsening of the Windkessel function and increases in peripheral resistance, which ultimately drive the progression of SBP. Aortas of EC-Del1 or DEL-1–Fc–treated mice were less stiff compared with their respective controls. This is demonstrated by assessment of the aortic diameter-tension relationship, a parameter for assessing vessel stiffness (38), which showed that aortas of EC-Del1 or DEL-1–Fc–treated mice developed less passive tension compared with those of controls. These mice also had less aortic adventitial fibrosis and medial thickening along with more elastin and profoundly improved endothelial function, which are additional indications that these mice had more elastic vessels with better endothelial function than their respective controls. Based on this, we conclude that DEL-1 did not interfere with vasoconstrictive effects and hence was unable to prevent the increase in SBP during the first week of hypertension induction. However, because DEL-1 prevented aortic remodeling and stiffness and endothelial dysfunction, it abrogated progression of SBP after the first and up to the fourth week of hypertension.

Along with arterial remodeling, hypertension is a major risk factor for development of cardiac remodeling, resulting in concentric hypertrophic cardiomyopathy with coronary microvascular dysfunction accompanied by metabolic adaptations. If untreated, these pathological changes may lead to cardiac failure (3–5). Here, we demonstrate that DEL-1 also protects from cardiac remodeling. This is supported by echocardiographic findings showing that, compared with their WT controls, EC-Del1 mice have higher LV diameter and end-diastolic volume as well as better systolic function shown by higher stroke volume. Histological assessment showed that EC-Del1 or Ec-DEL-1–Fc–treated mice had higher LV lumen-to-wall ratios with less cardiomyocyte cross-sectional area along with less interstitial and coronary fibrosis. EC-Del1 mice also showed better coronary perfusion by demonstrating improved coronary flow responses to adenosine and reactive hyperemia compared with WT littermates. It is notable that after ANGII infusion, WT mice had extremely worsened response to adenosine, which was accompanied by a severe decrease in mRNA levels of adenosine receptor types A2A and A2B. These receptors are major mediators of coronary vasodilation in response to adenosine (39). These findings are consistent with previous studies showing that ANGII worsens adenosine-induced vasodilation by diminishing adenosine receptor expression (39). However, it is not fully understood how ANGII reduces adenosine receptor expression. Because EC-Del1 mice showed better responses to adenosine and higher A2A and A2B receptor expression, antiinflammatory effects of DEL-1 might have at least partly contributed to higher expressions of these receptors. Nevertheless, the better coronary response to adenosine in EC-Del1 mice can be explained by higher expression of adenosine receptors, especially types A2A and A2B. Worsened coronary dilatation to adenosine and flow stop, beside diminished expression of adenosine receptors, can be a result of cardiac and coronary vessel remodeling. Increases in cardiomyocyte cross-sectional area and deposition of collagen in the extracellular matrix exert increased extravascular pressure, limiting the expansion of vessel radius and therefore limiting its dilatation. Moreover, perivascular fibrosis seen after ANGII infusion can stiffen coronary vessels and further hinder their dilatory capacity. This is especially evident in coronary dilatation after flow stop because the dilatation is not mediated only via adenosine and its receptors, but is a result of several mechanisms, including metabolic, endothelial NO, and myogenic responses. Therefore, better dilatory response of coronary vessels to adenosine and especially to flow stop in ANGII-infused EC-Del1 mice can be attributed both to higher expression of adenosine receptors and blunted cardiac and coronary vessel remodeling. Worsened coronary perfusion during cardiac hypertrophy leads to myocardial hypoxia and forces cardiomyocyte metabolic adaptation by a switch to increased glucose metabolism (4). This was evidenced in ANGII-infused WT mice, which showed higher lactate and malate levels in effluents of perfused hearts, whereas EC-Del1 mice had lower levels of these metabolites. These findings clearly demonstrate that DEL-1 improved cardiac parameters and coronary perfusion and blunted the increased reliance on glucose metabolism. Together, the findings in EC-Del1 mice and in therapeutic studies clearly demonstrate that DEL-1 attenuates hypertension-induced cardiovascular remodeling and further progression of SBP.

In this study, we also provide mechanistic proof of the actions of DEL-1 in ANGII-induced hypertension and cardiovascular remodeling. We reveal an action of DEL-1, a potent inhibitory effect of α_v_β_3_ integrin–dependent activation of latent MMP2. Studies in EC-Del1 mice and therapeutic studies clearly demonstrate low levels of active MMP2 in aortas and hearts without differences in latent pro-MMP2 levels after ANGII infusions. This suggests that DEL-1 did not affect expression of latent pro-MMP2, but inhibited α_v_β_3_ integrin–dependent activation of latent pro-MMP2 to active MMP2. The activation of latent pro-MMP2 is strictly regulated and hardly catalyzed by regular proteases. The activation rather depends on membrane α_v_β_3_ integrin, which localizes pro-MMP2 at the cell membrane and facilitates its catalytic cleavage by membrane protease MT1-MMP (25, 26). Because DEL-1 can bind to α_v_β_3_ integrin through its RDG motif (30), we propose that binding of DEL-1 to α_v_β_3_ integrin prevented this integrin-dependent activation of latent pro-MMP2. This is supported by 3 experimental setups using mice and human material, which demonstrate that, unlike DEL-1–Fc, mutant DEL-1–RGE–Fc, which does not interact with the α_v_β_3_ integrin, was unable to inhibit activation of latent pro-MMP2. Notably, a pharmacological inhibitor of α_v_β_3_ integrin acted like DEL-1–Fc and lowered active MMP2 levels without affecting pro-MMP2 levels. With this, we demonstrate for what we believe is the first time that DEL-1 can inhibit the activation of latent pro-MMP2 by blocking α_v_β_3_ integrin/pro-MMP2 interaction and can also inhibit its catalytic activation. We propose that the inhibitory effect of DEL-1 on activation of latent pro-MMP2 is an important mechanism of action of DEL-1 in the prevention of ANGII-induced cardiovascular remodeling because MMP2 is critically involved in hypertensive aortic and cardiac remodeling (23, 24). MMP2 is capable of degrading elastic fibers in aorta, leading to enhanced stiffening, whereas it also contributes to aortic remodeling by increasing endothelial permeability and promoting endothelial dysfunction, which may facilitate vascular inflammation (22, 40). Furthermore, a recent study in which authors used mice lacking MMP2 clearly demonstrates that these mice are protected from ANGII-induced aortic remodeling (23). Mice lacking MMP2 also showed less aortic monocyte/macrophage and T cell infiltration along with better endothelial function and decreased aortic stiffness. Importantly, these protective effects were observed in a mouse model lacking MMP2 in vascular cells. Furthermore, Diaz-Canestro et al. demonstrated that MMP2 knockdown by siRNA protected mice from age-dependent carotid stiffening and an increased elastin-to-collagen ratio, which was associated with increased eNOS phosphorylation and higher levels of cGMP (24). Therefore, inhibition of MMP2 by DEL-1 in aorta may represent an important mechanism of action in protection from at least vascular remodeling during hypertension. Although mice lacking MMP2 show protection from vascular stiffening, they still demonstrate cardiac inflammation and remodeling (23). DEL-1, however, was able to inhibit both cardiac and aortic inflammation and prevent cardiovascular remodeling. Based on this, we propose that, along with inhibition of pro-MMP2 activation, direct antiinflammatory effects of DEL-1 may extend protection from cardiac remodeling.

It has been established that inflammation is a critical driving force in hypertension-induced cardiovascular remodeling and progression of hypertension (6–13). There are 2 major proinflammatory actions driving hypertensive cardiovascular remodeling and hypertension: leukocyte recruitment into cardiovascular tissues followed by T cell/IL-17–driven inflammation and, simultaneously, a destabilized/reduced antiinflammatory CD4^+^FoxP3^+^ Treg/IL-10 response (6–12, 15–17). Genetic antiinflammatory approaches, such as depletion of T cells in RAG1-KO mice or adoptive transfer of Tregs along with substitution of IL-10, were shown to protect from ANGII-induced cardiovascular remodeling (6–12, 15–17). Previously, we showed that DEL-1 exerts its potent antiinflammatory actions via 3 major mechanisms: it inhibits β_2_ integrin–dependent recruitment of leukocytes and promotes resolution of inflammation via α_v_β_3_ integrin–dependent macrophage efferocytosis and induction of antiinflammatory Treg response (35, 36). Here, we demonstrate that in all of our 4 mouse experimental models, EC-Del1 or DEL-1–Fc–treated, but not mutant DEL-1–RGE–Fc–treated, mice were protected from hypertension-induced cardiovascular inflammation by infiltration of fewer CD45^+^ leukocytes, TCR-β^+^, and CD45^+^IL-17^+^ leukocytes in aorta and heart. Moreover, DEL-1–Fc injections during hypertension resulted in higher numbers of CD25^+^FoxP3^+^ Tregs and increased IL-10 compared with injections of DEL-1–RGE–Fc. The latter finding is consistent with our previous report demonstrating that DEL-1 induced a Treg response via an α_v_β_3_/RUNX1/FoxP3 mechanistic pathway (35). Finally, as described above, only injection of DEL-1–Fc, and not DEL-1–RGE–Fc, was able to abrogate cardiovascular remodeling and progression of hypertension. This indicates that antiinflammatory actions of DEL-1 and protection from cardiovascular remodeling were α_v_β_3_ integrin dependent. Therefore, α_v_β_3_ integrin–dependent actions, such as stabilization of the Treg/IL-10 response and inhibition of pro-MMP2 activation, are of critical importance. This might be true because hypertension-induced cardiovascular inflammation and organ damage are associated with loss of Treg/IL-10 responses and overactivation enhanced activation of MMP2 (13, 15, 17, 18, 23, 41). We want to point out that, although mutant DEL-1–RGE–Fc is unable to mediate α_v_β_3_ integrin–dependent actions of DEL-1, it is still able to bind to β_2_ integrins and might be able to inhibit recruitment of leukocytes (33). However, DEL-1–RGE–Fc was unable to suppress cardiovascular inflammation and organ damage. This indicates that the direct inhibition of leukocyte recruitment alone is most likely not sufficient to secure overall effective antiinflammatory actions of DEL-1 and protect from cardiovascular remodeling. Therefore, we propose that the actions of DEL-1, which are mediated by interaction with the α_v_β_3_ integrin, ensure efficient antiinflammatory actions and protection from hypertensive cardiovascular remodeling and progression of hypertension.

Efferocytosis is another α_v_β_3_ integrin–dependent inflammation-resolving action of DEL-1 (36). Although DEL-1–mediated efferocytosis was not addressed in our hypertension models, it is unlikely that this action is relevant, at least in our EC-Del1 model. Previously, we showed that DEL-1–dependent promotion of efferocytosis and resolution of inflammation depend on cell-specific expression of DEL-1 in a model of periodontitis. While EC-Del1, the model employed here, does not promote efferocytosis, macrophage-specific expression of DEL-1 resolves inflammation by efferocytosis (36). However, we cannot rule out the contribution of efferocytosis in therapeutic studies because injection of DEL-1–Fc is a different mode of intervention than overexpression of DEL-1 and might have, at least partly, acted via efferocytosis. Based on this, we propose that, in the model of hypertension employed here, the major mechanisms of action of DEL-1 were mediated via α_v_β_3_ integrin. These actions include inhibition of pro-MMP2 activation and stabilization of antiinflammatory Treg/IL-10 response. These α_v_β_3_ integrin–dependent actions led to suppression of inflammation and ameliorated remodeling in cardiovascular tissues and also blunted progression of hypertension. Whereas inhibition of pro-MMP2 activation might have been sufficient to protect from aortic remodeling and progression of hypertension, additional direct antiinflammatory actions of DEL-1 were most likely critical to ensuring protection from cardiac remodeling.

In conclusion, as summarized in Figure 15H, our findings provide compelling evidence that DEL-1 is a potent factor in attenuating ANGII- or DOCA-salt–induced hypertensive aortic and cardiac remodeling and, consequently, progression of hypertension. In this context, DEL-1 may act via a combination of mechanisms including, but not limited to, stabilization of Treg/IL-10 responses and inhibition of activation of latent pro-MMP2, which were associated with decreased inflammatory cell recruitment. Given the potent effects seen after therapeutic application of DEL-1–Fc, we specifically propose that DEL-1 is an attractive potential therapeutic agent against hypertension-induced cardiovascular remodeling. These findings greatly widen the horizon of potent immunomodulating effects of DEL-1 in inflammation-driven diseases.

## Methods

### Mice: induction of hypertension and cardiovascular remodeling.

We used DEL-1 transgenic mice (EC-Del1) with endothelial-specific overexpression of DEL-1 (31, 42, 43) and their WT littermates. Hypertension and cardiovascular remodeling were induced by employing 2 models of hypertension, infusion of 1.5 mg/kg/d of ANGII (MilliporeSigma) for up to 4 weeks using osmotic minipumps (Alzet, model 1001) and implantation of a DOCA-salt pellet (50 mg DOCA + 1% salt in drinking water) for up to 3 weeks. The pumps and the DOCA pellets were implanted subcutaneously at the age of 12 weeks under anesthesia with 2% isoflurane. Control mice were infused with vehicle (NaCl) or implanted with placebo pellets to control effects of ANGII and DOCA, respectively. Mice were sacrificed under deep anesthesia with a combination of 270 mg/kg ketamine and 30 mg/kg xylazine.

### Therapeutic studies in mice.

Therapeutic efficacy of DEL-1 in ANGII-induced (infused for 2 weeks) hypertension and cardiovascular remodeling was tested by retroorbital injection of recombinant soluble DEL-1–Fc in 12-week-old WT mice. Thirty micrograms of DEL-1 fused with an Fc receptor was administered per injection per mouse in the first therapeutic study, whereas 20 μg per injection was used in the second and third therapeutic studies. Recombinant DEL-1–Fc was produced as previously described (33). Along with DEL-1–Fc protein with an RGD motif, by which it binds to α_v_β_3_ integrin (30), a mutant form of the protein was used with an altered RGE motif (referred to throughout the text as DEL-1–RGE–Fc), which is unable to bind to α_v_β_3_ integrin and affect its function (35, 36). Three therapeutic studies were performed, one preventive, starting with 1 injection before the start of ANGII infusions and injections every second day after starting the infusions (Figure 6). In this study, the last injection was done at the 12th day of ANGII infusion. In two other therapeutic studies, interventions with injections started after established hypertension at the sixth or tenth day of ANGII pump or DOCA pellet implantation (after established hypertension; Figure 9 and Figure 12), respectively, followed by injections up to the 12th (ANGII) and 18th (DOCA) days. A total of 7 (ANGII) or 8 (DOCA) injections were performed. As control (placebo) groups, mice were injected with Fc.

### Experiments on human isolated aorta.

Human thoracic aorta was donated after surgical removal of aortic aneurysm. Aortic sections were stored in the vessel protection solution Tiprotec (F. Köhler Chemie), which preserves cell function of the vessel wall for at least 24 hours (44). Only the nondilated part of resected aortic segments was prepared and used for experiments. As described in Figure 13, the segments were divided (in approximately 5 mm long sections) and placed in a 12-well plate containing supplemented DMEM (Thermo Fisher). Aortic segments were pretreated with plasma (diluted in the medium) of WT mice previously injected with Fc, DEL-1–Fc, or DEL-1–RGE–Fc or plasma of EC-Del1 mice alone for 30 minutes, followed by stimulation with 1 μmol/L ANGII/ET1 for 8 hours. After pretreatments and stimulations, aortic segments were collected for in situ zymography and enzyme assays (described below).

### In situ zymography and MMP2/Furin/MT1MMP assays.

In situ gelatin zymography was performed for assessment of gelatinolytic activity of MMP2 in an 8 μM thick aortic tissue section fixed in zinc-based fixative (0.1 M Tris pH 7.4, 36.7 mM ZnCl_2_, 27.3 mM ZnAc_2_, 0.63 mM CaAc_2_) and embedded in paraffin (45). After deparaffinization in xylene, the tissue sections were rehydrated gradually in alcohol baths (100%, 3 × 2 minutes; 96%, 2 × 2 minutes; 70%, 2 minutes; 40%, 2 minutes, H_2_O, 2 minutes). Fluorogenic MMP2 substrate (0.1 % DQ gelatin, D12054, Thermo Fisher), which becomes fluorescent upon catalytic cleavage by MMP2, was added to the tissue sections and incubated in a dark humid chamber at 37°C for 20 hours. The sections were then fixed in 4% neutral-buffered formalin for 10 minutes in the dark, followed by washing in PBS baths (2 × 5 minutes). Finally, the tissue sections were mounted with glycerol containing DAPI to counterstain the nuclei and imaged with fluorescent microscopy (ApoTome, Zeiss). Because MMP2 is a zinc-dependent enzyme, some sections were incubated with fluorogenic MMP2 substrate mixed with 1 mmol/L EDTA to control for specific MMP2 activity. Fluorogenic signal intensity as a value for MMP2 activity was quantified using ImageJ, version 1.52a (NIH). Furin activity in human aortic extracts was assessed using the fluorogenic substrate Pyr-Arg-Thr-Lys-Arg-AMC (MerckMillipore), as previously described (26). A microplate reader (BMG Labtech) was used to detect fluorescence at 365 nm excitation and 450 nm emission. Enzymatic activities of MMP2 and MT1-MMP in human aortic tissue extracts were quantified by specific enzyme assay kits (Abcam, ab139447 and ab 139454) according to the user manual.

### Echocardiography.

Echocardiography in mice was performed under anesthesia with 2% isoflurane using the Fujifilm Visualsonics Vevo 3100 (with MX 400 transducer) echocardiography device. Obtained images were analyzed using Vevo Lab, version 2.1.0, software. The following parameters on the long axis of the LV in B and M modes were analyzed: end-diastolic diameter and volume, end-systolic diameter and volume, end-diastolic and end-systolic ventricular wall thickness.

### Isolated Langendorff heart experiments and metabolomics.

Coronary vessel function was assessed on isolated hearts, which were perfused with Krebs-Henseleit perfusion buffer, as previously performed (46). The buffer was delivered through a cannula inserted in the ascending aorta, allowing perfusion of the coronary vessels by creating a retrograde flow in the aorta. Coronary flow reserve was then tested as reactive hyperemia (evoked by stopping the flow for 20 seconds) or during infusion of 1 μmol/L adenosine. As perfusate drained as an effluent from the coronary circulation and dripped from the apex of the heart, the effluent was collected and targeted metabolomics was performed employing liquid chromatography–mass spectrometry (LC-MS) to assess metabolites of the TCA cycle.

### Histological stainings and morphometry.

Remodeling of thoracic aorta was evaluated by assessment of adventitial collagen, elastin area, and medial thickness employing sirius red, elastica van Gieson, and H&E stainings, respectively. Cardiovascular remodeling was evaluated by assessment of LV lumen-to-wall ratio and cardiomyocyte cross-sectional area after H&E staining as well as interstitial and perivascular epicardial coronary collagen after sirius red staining. Histological stainings and morphometry were performed as previously described (45).

### Measurement of SBP.

Before and after induction of hypertension, SBP was measured weekly in conscious mice using the tail-cuff method with a blood-pressure recorder (UGO Basil), as performed previously (45). Mice were trained for the procedure for 2 weeks.

### Assessment of endothelial function and aortic stiffness.

Endothelial function was evaluated with assessment of ACh-mediated endothelium-dependent aortic relaxation with a Mulvany myograph (Power Lab/400, AD Instruments), as previously described (45). Along with ACh, SNP was used to assess endothelium-independent aortic relaxation. Vessels were first preconstricted with KCl (123.7 mmol/L), and then various concentrations of ACh and SNP were applied. A Mulvany myograph was also used to evaluate aortic stiffness by assessment of change in the passive diameter-tension relation, as described previously (38).

### Flow cytometry.

Aorta with perivascular fat as well as heart was digested by incubating tissues in collagenase D (1 mg/ml), fispase II (1 mg/ml), and DNAse I (0,1 mg/ml) in RPMI medium. Tissues were cut into small pieces and vigorously shaken for 20 minutes at 37°C. After passing via cell strainer (40 μm), the enzymatic reaction was blocked by PBS containing EDTA (2 mM) and FBS (2%). After centrifugation at 400*g*, cells were resuspended in FACS buffer and stained with their respective antibodies: CD45, eBioscience, clone 30F11, catalog 4277450; TCR-β, BioLegend, catalog 109222; CD4, MACS Miltenyi Biotec, catalog 130-102-619; CD8, Miltenyi Biotec, catalog 130-102-468; CD25, BD Biosciences — Pharmingen, catalog 553075; and FoxP3, eBioscience, catalog 14-5773-82. For intracellular stainings, part of the cell suspension was used to culture cells in 24-well culture dishes in RPMI+10%FBS+1% penicillin/streptomycin medium. After 24 hours, the cells were stimulated with PMA/ionomycin containing brefeldin A and monensin (protein transport inhibitors) for 4 hours. To distinguish death cells, Zombie Aqua Fixable Viability Kit (BioLegend) was used prior to antibody staining with anti–IL-17 antibody (Thermo Fisher, catalog 17-7177-81). The cells were assessed with flow cytometer (BD FACSCanto II Cell Analyzer). Data were analyzed with FlowJo software (Tree Star, Inc.).

### Experiments on isolated mouse aorta and cultured human aortic SMCs.

Thoracic aorta was isolated from 12-week-old mice and placed in a 12-well plate containing supplemented DMEM (Thermo Fisher). Primary cells were purchased from PromoCell. Cells were cultured on a 12-well plate and stimulated with 1 μmol/L of ANGII and ET1 (MilliporeSigma) for 8 hours to induce latent pro-MMP2 expression and activation. Effective concentrations of ANGII and ET1 were chosen based on our previous reports (26, 27). Various experiments on isolated aorta and human aortic SMCs were performed, as described in Figure 11. The aorta and cells were pretreated with 0.1 μg/ml DEL-1–Fc or mutated DEL-1–RGE–Fc. As a control group, the cells were pretreated with the pharmacological inhibitor of α_v_β_3_ integrin cilengitide (1 μmol/L, Selleckchem). In other experiments, cells were pretreated with plasma of mice injected with either Fc, DEL-1–Fc, or DEL-1–RGE–Fc plasma of EC-Del1 mice for 30 minutes. After this, plasma was removed and cells washed gently once, followed by stimulation with 1 μmol/L ANGII/ET1 for 8 hours.

### Gelatin zymography.

The method was used to assess latent 72 kDa pro-MMP2 and 62 kDa active MMP2 in extracts of aorta and heart as well as in SMC supernatant, as previously described (26, 27). Briefly, 0.25 % gelatin copolymerized in 10% polyacrylamide gel was used as a substrate for MMP2. Five micrograms of protein was separated with electrophoresis, followed by incubation of gels in developing buffer. After 24 hours, gels were stained with 0.1 % Coomassie blue. Latent pro-MMP2 and active MMP2 were detected as white bands on homogenously stained gel. Images were taken with the PEQLAB Imager. Bands were quantified by densitometry using ImageJ, version 1.52a.

### Reverse transcription qPCR and ELISA.

Inflammatory and remodeling (hypertrophy, fibrosis) profiles in aorta and heart were evaluated by assessment of the mRNA or protein levels of various proinflammatory and remodeling markers. mRNA was isolated from homogenized tissues (Polytron) using an mRNA isolation kit (RNeasy Plus Universal Mini Kit, QIAGEN) according to the user’s manual. As previously described (44), 1 μg total mRNA was reverse transcribed using the iScript cDNA Synthesis Kit (no. 170-8891; Bio-Rad), and cDNA was amplified using the respective primers and SsoFast EvaGreen Supermix (no. 172-5240; Bio-Rad). Proinflammatory markers in tissue lysates were assessed using the V-Plex Proinflammatory Panel 1 Mouse Kit (K15048D-1, Mesoscale) according to the user’s manual.

### Statistics.

Results were analyzed with 1- or, when appropriate, 2-way ANOVA for multiple comparisons using GraphPad Prism, version 6.01, for windows, followed by Bonferroni’s post hoc test to adjust for multiple comparisons. Data in Figure 4, A–D, Figure 7, A–D, Figure 10, A–D, and Figure 13N were analyzed for repeated measures. Data are represented as mean ± SEM. A P value of less than 0.05 was considered significant.

### Study approval.

Experiments on human isolated aorta and animal studies were approved by the Ethics Committee of the University Hospital Dresden (approval EK26806201) and by the IRB and Landesdirektion Sachsen (TVV 63-2015, TVV 37-2019), respectively. All human subjects gave written, informed consent.

## Author contributions

I Kopaliani designed and supervised the work. AD and TC cosupervised the work. TF, MAO, AN, I Kopaliani, AS, FG, ACW, PS, I Kourtzelis, and MP conducted experiments. TF, MAO, AN, I Kopaliani, PM, and MP acquired data. TF, MAO, AN, I Kopaliani, and AD analyzed data. I Kopaliani, AD, VT, TC, and GH interpreted data. PS, I Kourtzelis, CH, VT, TK, XL, GH, TC, KM, and SMT provided reagents. I Kopaliani and AD wrote the manuscript. GH, TC, and VT edited the manuscript.

## Supplementary Material

Supplemental data

## Version 1 02/08/2022

In-Press Preview

## Version 2 03/15/2022

Electronic publication
