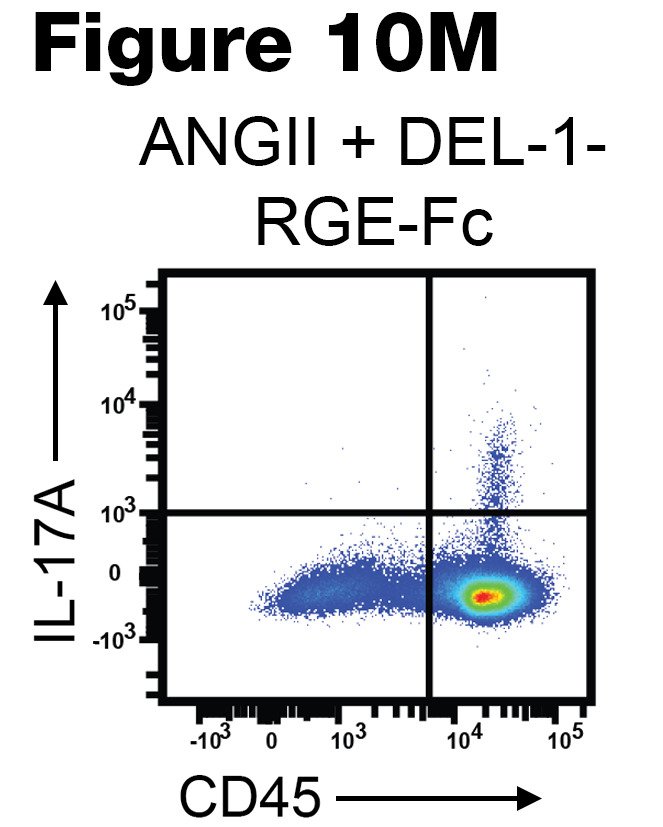# Developmental endothelial locus-1 protects from hypertension-induced cardiovascular remodeling via immunomodulation

**DOI:** 10.1172/JCI181599

**Published:** 2024-05-01

**Authors:** Theresa Failer, Michael Amponsah-Offeh, Aleš Neuwirth, Ioannis Kourtzelis, Pallavi Subramanian, Peter Mirtschink, Mirko Peitzsch, Klaus Matschke, Sems M. Tugtekin, Tetsuhiro Kajikawa, Xiaofei Li, Anne Steglich, Florian Gembardt, Annika C. Wegner, Christian Hugo, George Hajishengallis, Triantafyllos Chavakis, Andreas Deussen, Vladimir Todorov, Irakli Kopaliani

Original citation: *J Clin Invest*. 2022;132(6):e126155. https://doi.org/10.1172/JCI126155

Citation for this corrigendum: *J Clin Invest*. 2024;134(9):e181599. https://doi.org/10.1172/JCI181599

The authors recently became aware of 3 errors in the original publication. In Figure 7H, the Vehicle+Fc flow cytometry panel was incorrect. In Figure 9M, the ANGII+DEL-1-FC panel was incorrect. In Figure 10M, the ANGII+DEL-1-RGE-Fc was incorrect. The correct images, provided from the original source data, are shown below.

The authors regret the errors.

## Figures and Tables

**Figure:**
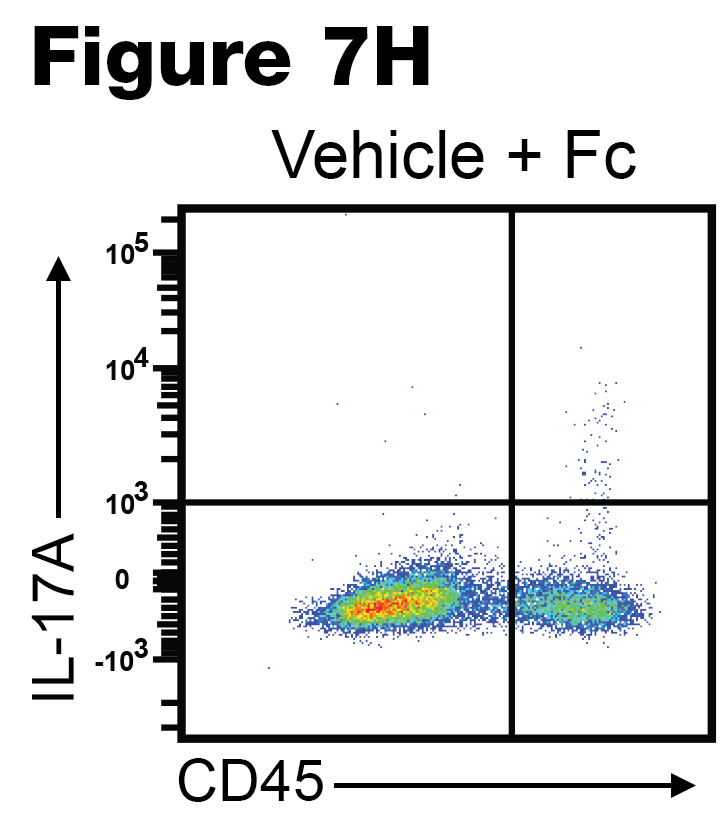


**Figure:**
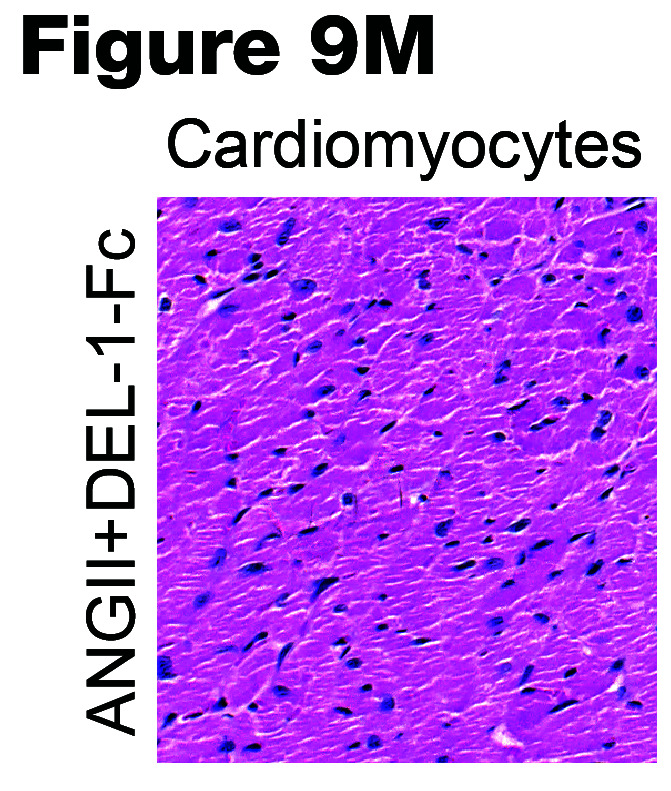


**Figure:**