# Supplementary material for: Developmental endothelial locus-1 protects from hypertension-induced cardiovascular remodeling via immunomodulation
Source: J Clin Invest. 2022 Mar 15;132(6):e126155. doi: 10.1172/JCI126155 (PMC8920341; doi:10.1172/JCI126155)

Supplemental Figure 1. EC-Del1 mice have low inflammatory profile in aorta. mRNA levels of aortic fibrotic **(A-C)**, pro- **(E-M)** and anti-inflammatory **(N)** markers. mRNA levels of Del-1 **(O)** (n=6-8 mice per group for all experiments). Data represent mean ± SEM. **P*<0.05, ***P*<0.01 analyzed by two-way ANOVA with Bonferroni post hoc test to adjust for multiple comparisons.

Supplemental Figure 2. EC-Del1 mice have low inflammatory profile in heart. mRNA levels of cardiac fibrotic **(A-B)**, hypertrophic **(C-F)**, pro- **(G-M**) and anti-inflammatory **(N)** markers. mRNA levels of Del-1 **(O)** (n=6-8 mice per group for all experiments). Data represent mean ± SEM. **P*<0.05, ***P*<0.01 analyzed by two-way ANOVA with Bonferroni post hoc test to adjust for multiple comparisons.

Supplemental Figure 3. DEL-1-Fc treated mice have low inflammatory profile in aorta. mRNA levels of aortic fibrotic **(A-C)**, pro- **(D-K)** and anti-inflammatory **(L)** markers (n=6 mice per group). Data represent mean ± SEM. **P*<0.05, ***P*<0.01 analyzed by one-way ANOVA with Bonferroni post hoc test to adjust for multiple comparisons.

Supplemental Figure 4. DEL-1-Fc treated mice have low inflammatory profile in aorta. mRNA or protein levels of cardiac fibrotic **(A-B)**, hypertrophic **(C-F)**, pro- **(G-K**) and anti-inflammatory **(L)** markers (n=6 mice per group). Data represent mean ± SEM. **P*<0.05, ***P*<0.01 analyzed by one-way ANOVA with Bonferroni post hoc test to adjust for multiple comparisons.

Supplemental Figure 5. DEL-1-Fc, but not DEL-1-RGE-Fc treated mice have low inflammatory profile in aorta. mRNA or protein levels of aortic fibrotic **(A-C)**, pro- **(D-K)** and anti-inflammatory **(L)** markers (n=6 mice per group). Data are means ± SE. **P*<0.05, ***P*<0.01 analyzed by one-way ANOVA with Bonferroni post hoc test to adjust for multiple comparisons.

Supplemental Figure 6. DEL-1-Fc, but not DEL-1-RGE-Fc treated mice have low inflammatory profile in heart. mRNA or protein levels of cardiac fibrotic **(A-B)**, hypertrophic **(C-F)**, pro- **(G-K**) and anti-inflammatory **(L)** markers (n=6 mice per group). Data are means ± SE. **P*<0.05, ***P*<0.01 analyzed by one-way ANOVA with Bonferroni post hoc test to adjust for multiple comparisons.

Supplemental Figure 1

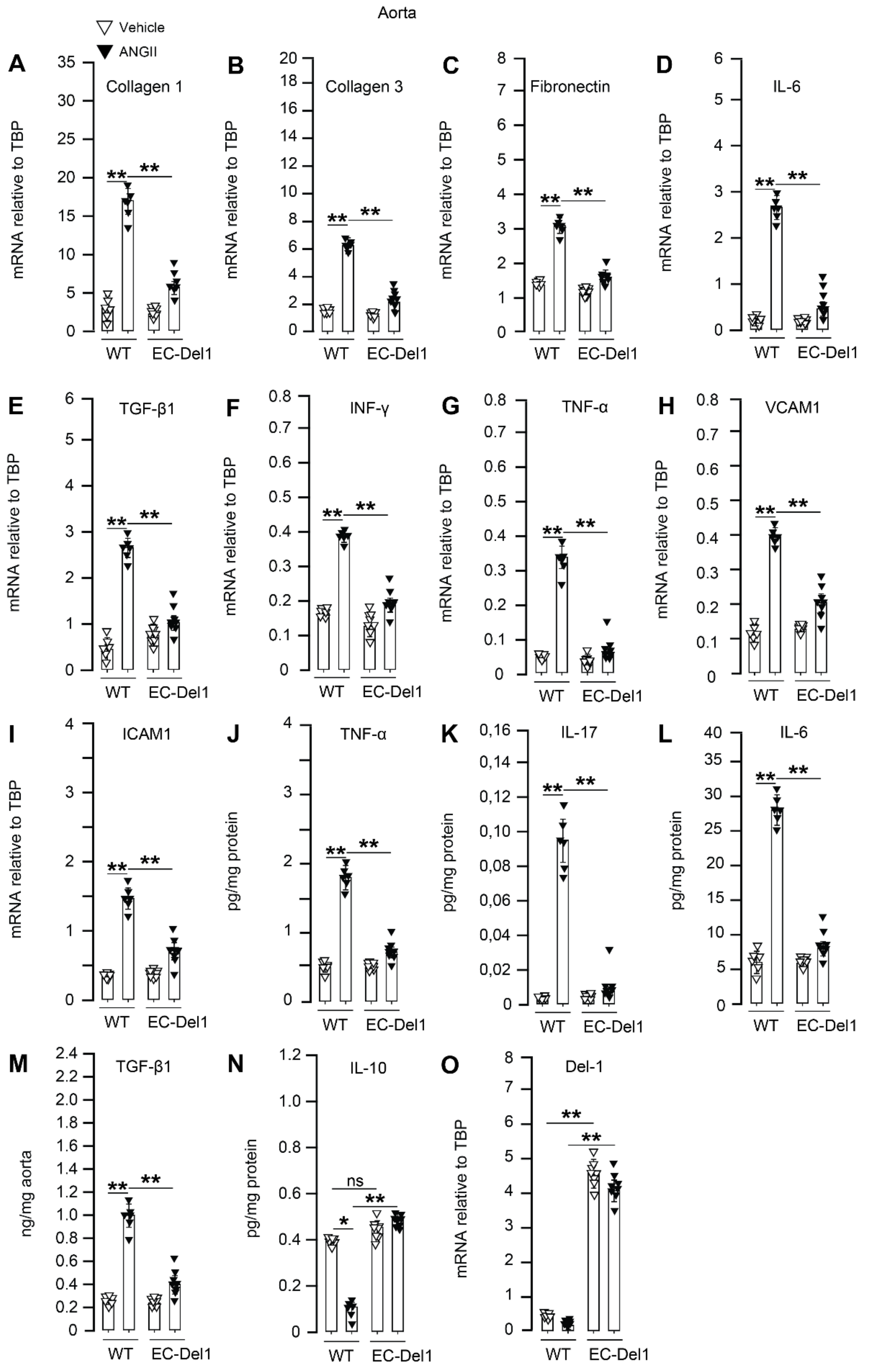

Supplemental Figure 2

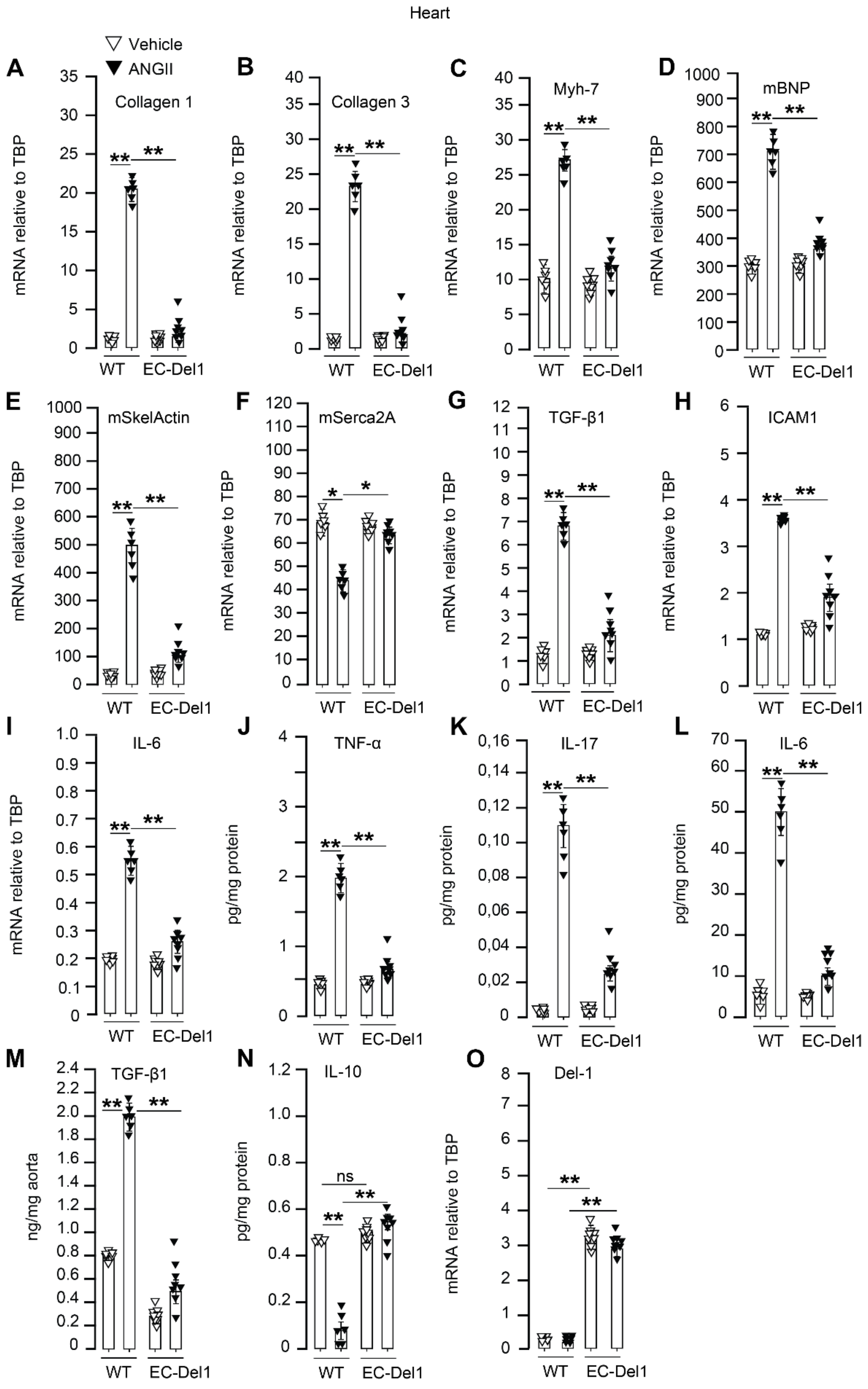

Supplemental Figure 3

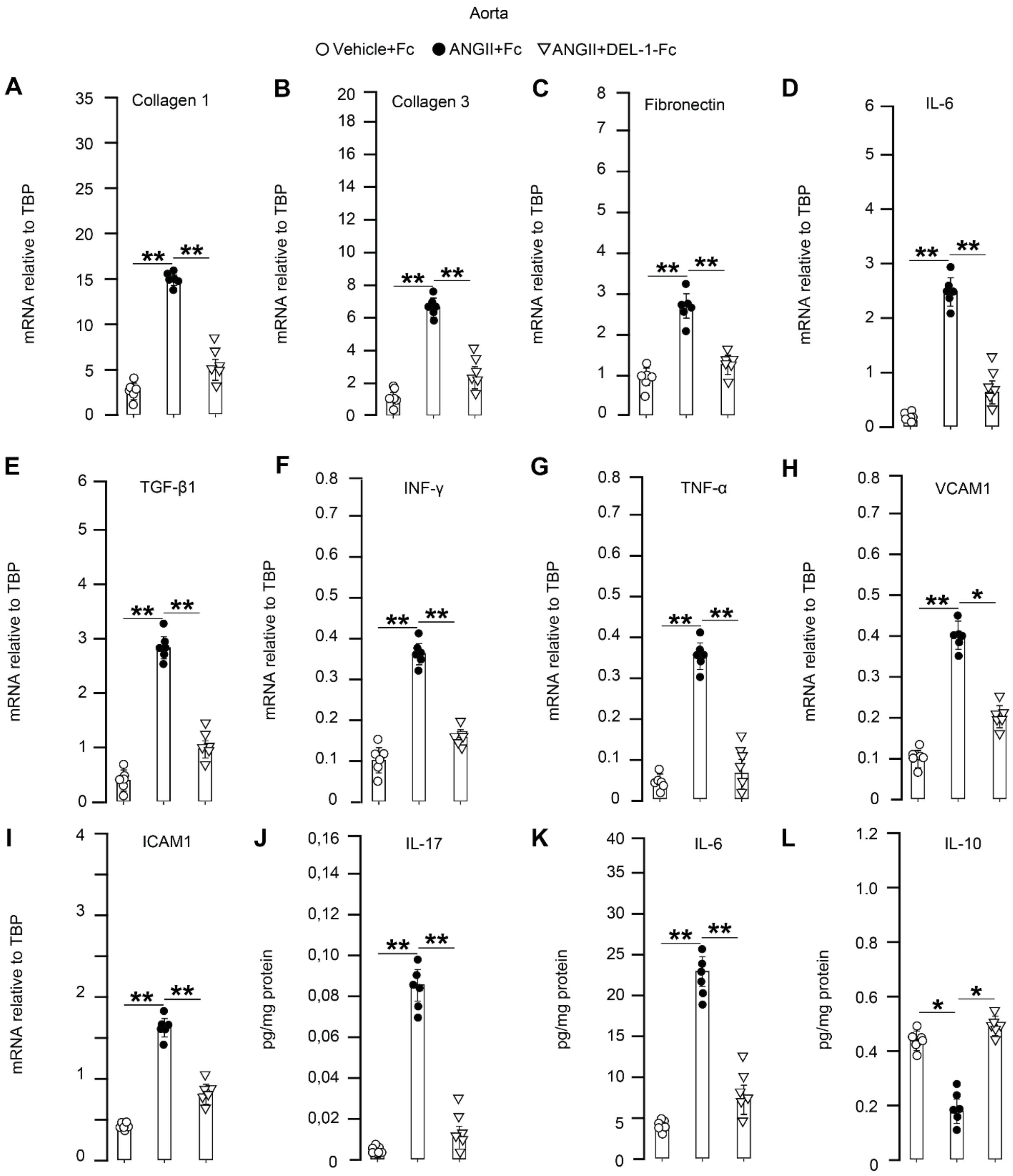

Supplemental Figure 4

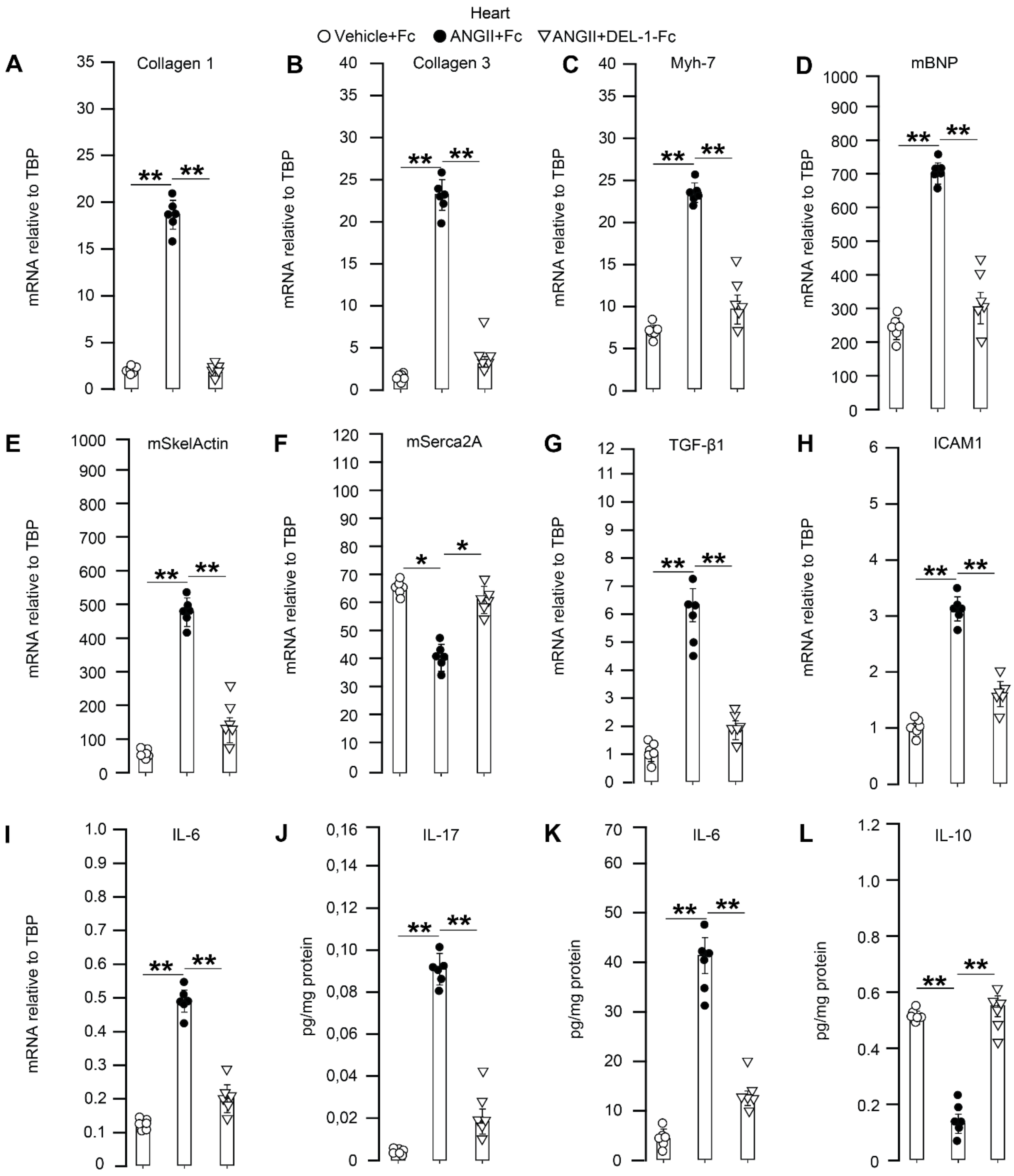

Supplemental Figure 5

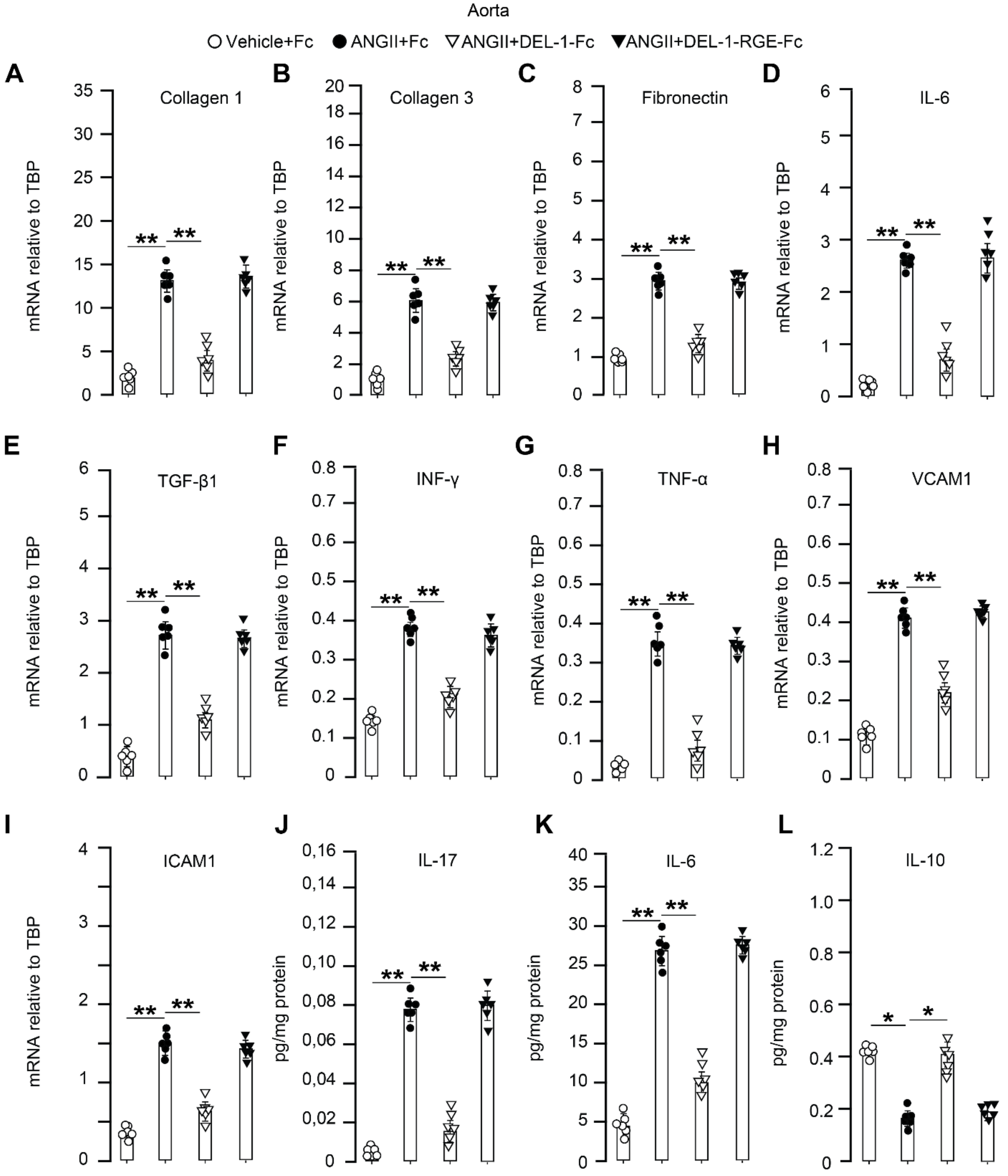

Supplemental Figure 6

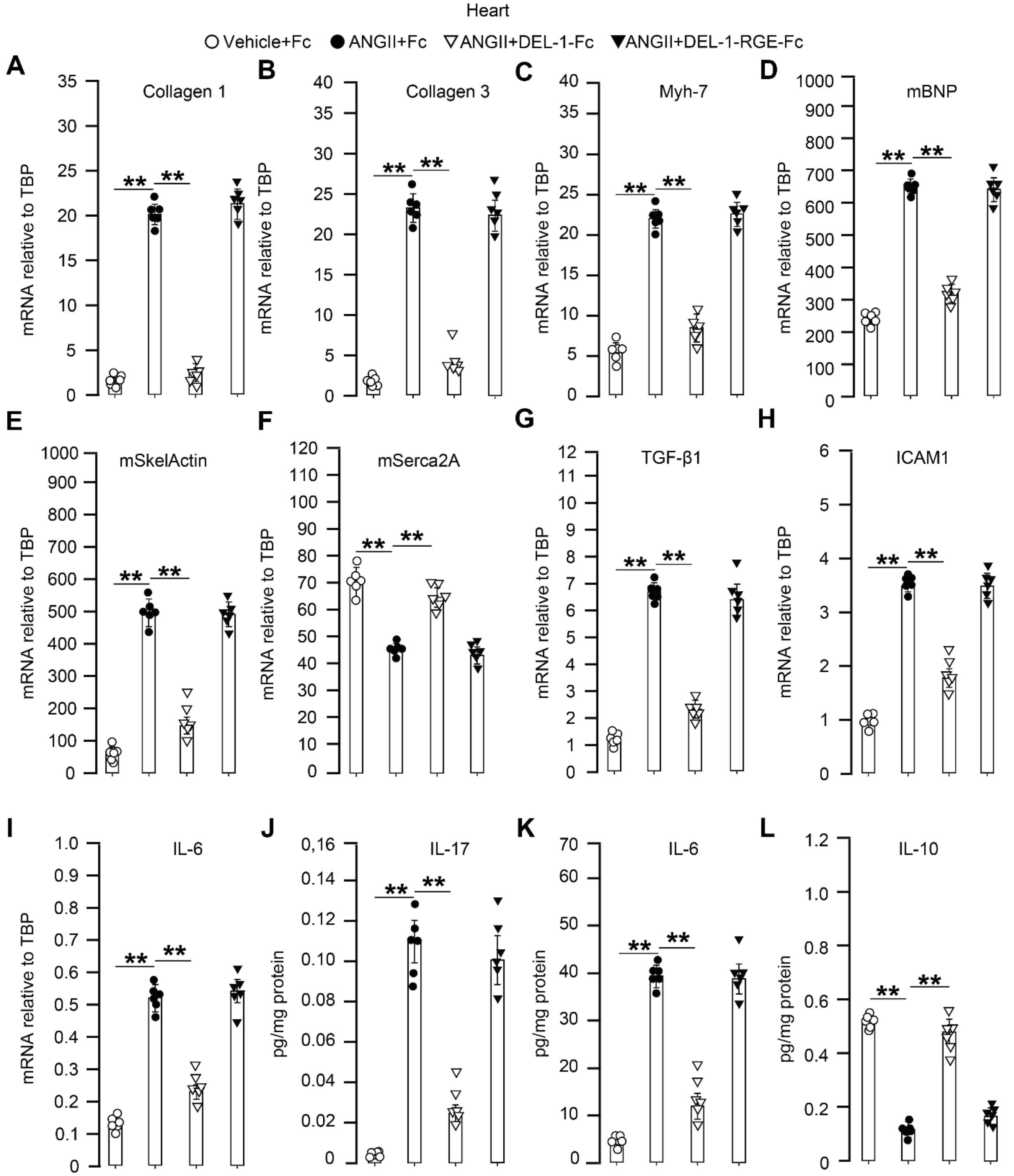

Supplement: Supplemental data [file jci-132-126155-s016.pdf]
